# Plk4 Phosphorylates Ana2 to Trigger Sas6 Recruitment and Procentriole Formation

**DOI:** 10.1016/j.cub.2014.08.061

**Published:** 2014-11-03

**Authors:** Nikola S. Dzhindzhev, George Tzolovsky, Zoltan Lipinszki, Sandra Schneider, Ramona Lattao, Jingyan Fu, Janusz Debski, Michal Dadlez, David M. Glover

**Affiliations:** 1 Department of Genetics, University of Cambridge, Cambridge CB2 3EH, UK; 2 Laboratory of Mass Spectrometry, Institute of Biochemistry and Biophysics, Polish Academy of Sciences, 02-106 Warsaw, Poland

## Abstract

Centrioles are 9-fold symmetrical structures at the core of centrosomes and base of cilia whose dysfunction has been linked to a wide range of inherited diseases and cancer [1]. Their duplication is regulated by a protein kinase of conserved structure, the *C. elegans* ZYG-1 or its Polo-like kinase 4 (Plk4) counterpart in other organisms [2, 3, 4]. Although Plk4’s centriolar partners and mechanisms that regulate its stability are known, its crucial substrates for centriole duplication have never been identified. Here we show that *Drosophila* Plk4 phosphorylates four conserved serines in the STAN motif of the core centriole protein Ana2 to enable it to bind and recruit its Sas6 partner. Ana2 and Sas6 normally load onto both mother and daughter centrioles immediately after their disengagement toward the end of mitosis to seed procentriole formation. Nonphosphorylatable Ana2 still localizes to the centriole but can no longer recruit Sas6 and centriole duplication fails. Thus, following centriole disengagement, recruitment of Ana2 and its phosphorylation by Plk4 are the earliest known events in centriole duplication to recruit Sas6 and thereby establish the architecture of the new procentriole engaged with its parent.

## Highlights

• Plk4 phosphorylates Ana2 at essential residues in its conserved STAN motif
• Plk4 phosphorylation triggers the direct interaction of Ana2 with Sas6
• Ana2 phosphorylated by Plk4 recruits Sas6 to centrioles at the end of mitosis
• A phospho-null Ana2 mutant fails to recruit Sas6 and duplicate centrioles

## Teaser

Plk4 kinase triggers centriole duplication. However, its critical substrate has never been found. Here we show that *Drosophila* Plk4 phosphorylates four conserved serines in the STAN motif of the core centriole protein Ana2. This enables Ana2 to bind and recruit Sas6 toward the end of mitosis to trigger procentriole formation in the next cell cycle.

## Results and Discussion

**Figure 1:**
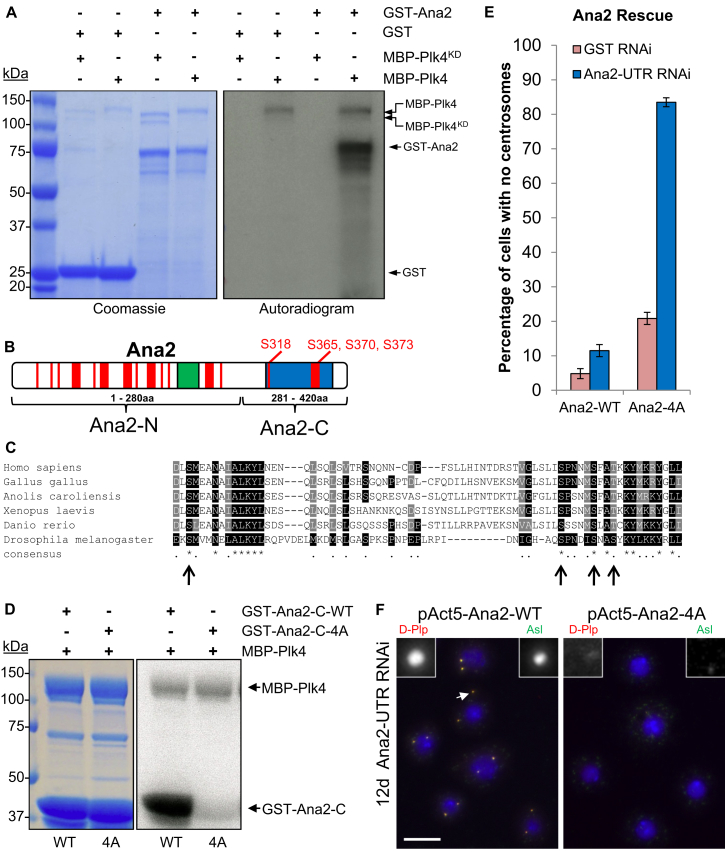
Plk4 Phosphorylates Ana2 at Four Essential Serine Residues in the Conserved STAN Motif (A) GST-Ana2 or GST were incubated with active (MBP-Plk4) or inactive (MBP-Plk4^KD^) Plk4 in the presence of γ-[^32^P]ATP and run on SDS-PAGE (Coomassie), photographed, dried, and directly subjected to autoradiography. (B) Plk4 phosphorylation sites (red bars) revealed by mass spectrometry (see Figure S1G) of which four serines are in the conserved STAN motif (blue box). Green box is predicted coiled-coil. (C) Alignment of STAN motifs in Ana2 orthologs showing the four conserved Plk4 phosphorylation sites (arrows). (D) Plk4 phosphorylates C-terminal portion (indicated in B) of wild-type Ana2 (GST-Ana2-C-WT) but not mutant with alanine substitutions at the four mapped Plk4 sites (GST-Ana2-C-4A). (E) Cell lines overexpressing either Ana2-WT or Ana2-4A from the *actin5c* promoter were treated with either control dsRNA (GST-RNAi) or dsRNA targeting the UTRs of endogenous Ana2 (Ana2-UTR RNAi). Cells with no centrosomes were scored after three rounds (12 days in total) of dsRNA treatment. Error bars represent SEM. (F) Micrographs from (E) showing centrosomes revealed by D-Plp and Asl costaining in the two cell lines following Ana2-UTR RNAi. Insets show D-Plp and Asl in monochrome from one pole (indicated by a white arrow). DNA is stained with DAPI (blue). Scale bar represents 10 μm.

We now have quite detailed knowledge of the partners of the Plk4 family of kinases at centrioles. In *C. elegans,* ZYG-1 is targeted to centrioles by SPD-2 [5, 6] whereas in *Drosophila*, Asterless has this function [7]. Targeting in mammalian cells requires the respective counterparts of both proteins Cep192 and Cep152 [8, 9] that can each interact with Plk4’s cryptic polo-box domain. Procentriole formation can be initiated at multiple sites not only when Plk4 is overexpressed [3, 4, 10] or when its SCF-dependent proteolysis is prevented [11, 12], but also when expression of its targeting subunit is elevated [7]. Despite this extensive knowledge, the identity of Plk4’s critical substrate for centriole duplication has remained elusive. Several substrates of Plk4/ZYG-1 have been identified to date that include SAS-6 [13], Cep152 [14], and a component of γ-TuRC, Gcp6 [15], but it is not clear how phosphorylation of these proteins might affect centriole duplication. To address this question we chose to identify centriole proteins that could be phosphorylated by Plk4 in vitro. To this end we purified an active form of *Drosophila* Plk4 expressed in *E. coli* that was able to undertake known autophosphorylation [16, 17, 18, 19, 20] and was also active toward an artificial substrate (Figure S1A available online). We first tested whether this preparation of Plk4 would phosphorylate proteins found in the outer layers of the centriole [21, 22]. This revealed that Plk4 could weakly phosphorylate its partner protein, Asl (Figure S1B), and Cep97 (Figure S1C), a protein that complexes with the Cp110 centriole capping protein [23]. However, it could not phosphorylate the microtubule wall-associated protein, Sas4 [21, 24, 25, 26], (Figure S1B); Rcd4, a poorly characterized centriole duplication protein (Figure S1C); or Bld10/Cep135, a protein required for maintenance but not formation of the core centriole [27, 28] (Figure S1C). We then asked whether the core centriole proteins Ana2 and Sas6 might be substrates as both are essential for centriole duplication in *Drosophila* [29, 30] and their respective counterparts in *C. elegans*, SAS-5 and SAS-6, are immediately downstream of ZYG-1 in the recruitment hierarchy of centriole proteins in *C. elegans* [5, 6]. Strikingly, Plk4 could strongly phosphorylate Ana2 but not Sas6 (Figures 1A, S1D, and S1E), suggesting the possibility that Ana2 might be the Plk4 substrate that triggers centriole duplication.

To test the above hypothesis, we first mapped the sites on Ana2 phosphorylated by Plk4 in vitro and tested the significance of their modification. Mass spectrometric analysis revealed multiple Plk4 phosphorylation sites of which four serine residues (S318, S365, S370, and S373) (Figures 1B and 1C, arrows, Figure S1G) stood out because their total spectral counts (times a particular phospho-peptide was detected) were much higher than any others. Moreover, they seemed to be the only Plk4 sites in the C-terminal part of Ana2 as their mutation to alanine prevented phosphorylation by Plk4 in vitro (Figures 1D and S1F). Their functional importance was also suggested by their conservation within the STAN motif that characterizes Ana2 orthologs (Figure 1C) and the finding that phosphorylation of the same sites could be detected in vivo (see Figure S1G and legend). To test their biological significance, we asked whether Ana2 with alanine substitutions at these sites (Ana2-4A) would permit centriole duplication. For this purpose we first established two D.Mel-2 cell lines, stably expressing untagged versions of either wild-type Ana2 (Ana2-WT) or Ana2-4A that each lacked the UTRs of the endogenous gene. We then depleted endogenous Ana2 from these lines using dsRNA against its UTRs. Three 4-day treatments of control D.Mel-2 cells with ds *ana2-UTR* RNA led to complete loss of centrioles from 68% of cells (data not shown). By contrast, depletion of endogenous Ana2 from the line expressing the Ana2-WT transgene had no significant effect upon centriole number, indicating that it can fully substitute for the endogenous protein. However, expression of transgenic Ana2-4A not only failed to rescue endogenous Ana2 depletion, but also had a significant dominant-negative effect, an increased proportion of cells lacking centrioles following control-RNAi (Figures 1E and 1F). Together this demonstrates the functional importance of these four conserved serines in the STAN motif of Ana2 for centriole duplication.

**Figure 2:**
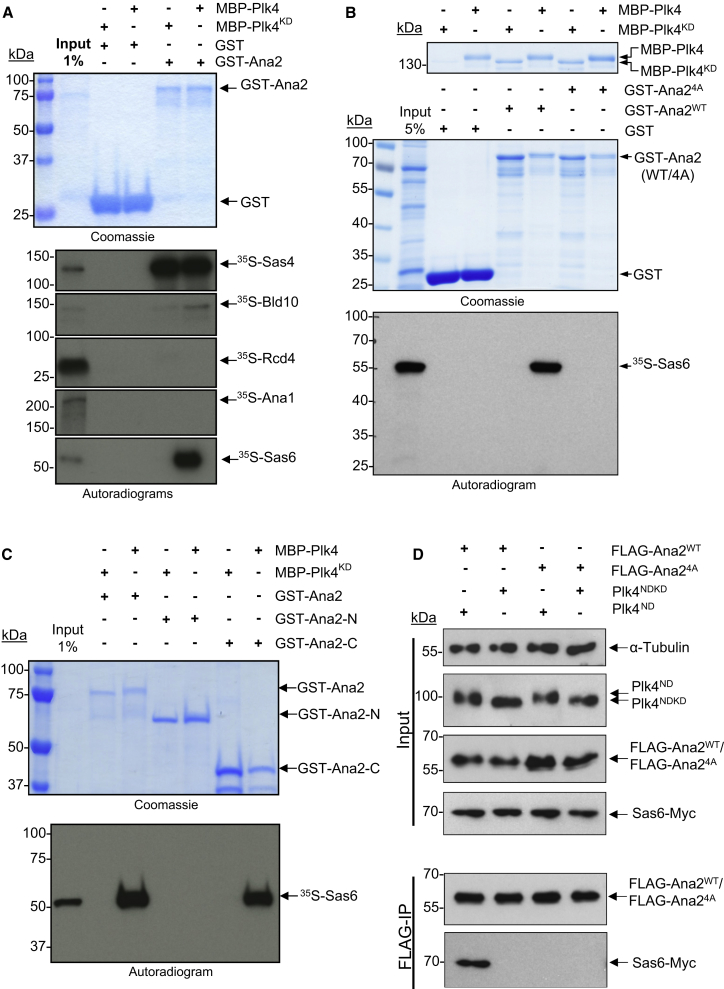
Plk4 Triggers a Direct, Phosphorylation-Dependent Interaction between Ana2 and Sas6 In Vitro and In Vivo (A) In vitro screen for differential interactions of centriolar proteins with Ana2 with and without phosphorylation by Plk4. GST-Ana2 treated with active (MBP-Plk4) or inactive (MBP-Plk4^KD^) Plk4 and incubated with ^35^S-Met-labeled Sas4, Bld10, Rcd4, Ana1, or Sas6 synthesized by in vitro transcription and translation. Affinity-purified complexes were analyzed by SDS-PAGE (Coomassie) followed by autoradiography. Note: Sas6 interacts with Plk4-prephosphorylated Ana2 but not with Ana2 treated with the inactive kinase. See Figure S2A for longer exposure showing interaction with Bld10. (B) GST, GST-tagged wild-type Ana2 (GST-Ana2-WT), or the four alanine substitution mutant (GST-Ana2-4A) were treated with either MBP-Plk4 or MBP-Plk4^KD^ and incubated in vitro with ^35^S-Met-labeled Sas6. The resulting complex was analyzed by SDS-PAGE (Coomassie) and autoradiography. Sas6 specifically interacts with Ana2, when the four conserved serines in the STAN motif are pre-phosphorylated by Plk4. (C) GST-Ana2, GST-Ana2-N (residues 1–280), or GST-Ana2-C (residues 281–420) incubated with either MBP-Plk4 or MBP-Plk4^KD^ and ^35^S-Met-labeled Sas6 and subjected to SDS-PAGE and autoradiography. C-terminal but not N-terminal part of Ana2 binds to Sas6 in vitro. (D) FLAG-tagged wild-type Ana2 (FLAG-Ana2^WT^) or the four alanine substitution mutant (FLAG-Ana2^4A^) were transiently cooverexpressed with Myc-tagged Sas6 (Sas6-Myc) and either the degron mutant (Plk4^ND^) or degron/kinase dead (Plk4^NDKD^) forms of Plk4 in D.Mel-2 cells. Input and anti-FLAG-immunoprecipitates were subjected to SDS-PAGE and Western blotting to reveal the indicated antigens.

We next considered whether phosphorylation of Ana2 by Plk4 might affect its interaction with other components of the centriole duplication machinery. To this end we loaded recombinant GST-Ana2 onto beads, incubated with either active or inactive (Plk4^KD^) kinase and then with ^35^S-methionine-labeled centriole proteins synthesized in vitro. We were unable to detect binding of Ana2 to either Rcd4 or Ana1; its binding to Sas4 showed no change; and its weaker binding to Bld10 showed a 3.5-fold increase in response to Ana2’s phosphorylation state (Figures 2A and S2A). However, Sas6 showed a dramatic increase in binding to Ana2 phosphorylated by Plk4 (Figures 2A and 2B). The C-terminal part of Ana2 containing the STAN motif was necessary and sufficient for this phospho-dependent interaction with Sas6 (Figure 2C), leading us to test the consequences of mutations at its four Plk4 sites. We found that the Ana2-4A mutant was unable to interact with Sas6 even after incubation with active Plk4 (Figure 2B), indicating that phosphorylation on these sites is required. When we mutated individual serines to alanines, the strength of the interaction was diminished, particularly with S370A mutant, but not completely abolished (Figure S2B). Thus Plk4 phosphorylation of Ana2 on all four residues is critical to mediate its interaction with Sas6 in vitro. To validate these findings in vivo, we cotransfected D.Mel-2 cells with Sas6-Myc and FLAG-Ana2 (either WT or 4A) and either active Plk4 mutated in its degron (Plk4^ND^) or inactive Plk4 (“nondegradable and kinase-dead” Plk4^NDKD^). Following FLAG-pulldown we could detect Sas6 associated with Ana2-WT but not Ana2-4A and only when coexpressed with the active form of Plk4 (Figure 2D). This verifies our in vitro findings that following phosphorylation by Plk4, Ana2 is able to interact with Sas6.

Sas6 provides a structural basis for centriole architecture; its oligomers adopt a 9-fold symmetrical arrangement to form the cartwheel structure of the procentriole [31, 32]. Stil (human Ana2) and hSas6 are the first proteins to follow Plk4 to a dot-like structure marking assembly of the procentriole [9, 22, 33]. Sas6 is essential for correct centriole structure in *Drosophila* although, unlike Plk4, its overexpression does not lead to proper centriole formation in eggs [34]. However, boosting expression of both Sas6 and Ana2 stimulates formation of multiple microtubule organizing centers in eggs [35] and tubular aggregates linked to disengaged centrioles in spermatocytes [36]. Interestingly, however, such Sas6 and Ana2 could be recruited to centrioles only if Plk4 were also overexpressed in spermatocytes leading to centriole overduplication [36]. These earlier findings might be accounted for if the phosphorylation of Ana2 by Plk4 triggered the first step in cartwheel formation by Sas6, leading to procentriole formation.

To address the above hypothesis, we first needed to examine the progressive recruitment of Ana2 and Sas6 to centrioles in their duplication cycle relative to the outer centriolar marker D-Plp. At mitotic entry, each centrosome comprises a pair of orthogonally *engaged* centrioles, which we refer to as mother and daughter, surrounded by peri-centriolar, microtubule-nucleating material. The daughter centriole is immature at this stage and matures during mitosis [37]. In *Drosophila* cells, the mother centriole is encircled by a D-Plp ring and during maturation, two “horns” of D-Plp progressively extend around the daughter to give a complete ring by metaphase/early anaphase. Once the D-Plp ring is complete, the paired centrioles *disengage* during late anaphase so that each newly born cell exits cytokinesis into G1 with two well-separated centrioles (Figure S3). At mitotic entry, Sas6 and Ana2 are both present in two discrete puncta, one in the center of the mother centriole, the other marking the daughter and yet to become encircled by D-Plp. When the mother and mature daughter disengage, they each have a single dot of Ana2 or Sas6 at their center. Then, in late anaphase/telophase, a second Ana2/Sas6 dot appears at the periphery of each physically separated centrioles, marking the site of procentriole formation (Figure S3).

**Figure 3:**
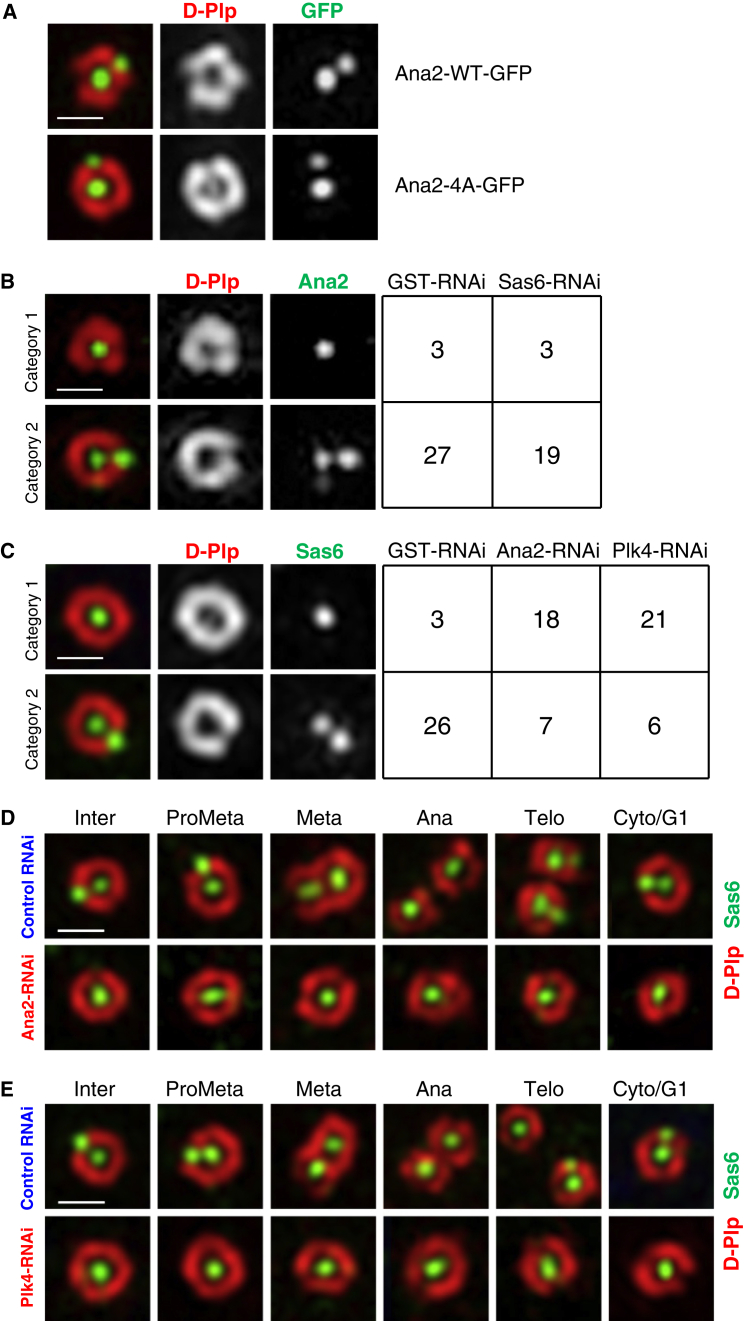
Plk4 and Ana2 Are Essential for Sas6 Loading onto Centrioles, while Ana2 Loading Is Independent of Sas6 (A) Structured illumination images of centrioles showing exogenous GFP-tagged wild-type Ana2 and its mutant form with alanine substitutions in the four serines phosphorylated by Plk4 (Ana2-WT-GFP and Ana2-4A-GFP, respectively) both associate with two distinct puncta in interphase centrioles (compare to endogenous Ana2; Figure S3). Note that endogenous Ana2 was not depleted in these experiments. (B) Categories of Ana2 localization in interphase centrioles following control (GST dsRNA) or Sas6 RNAi. (C) Categories of Sas6 localization following control (GST), Ana2, or Plk4 RNAi. Numbers of centrioles counted and imaged are shown on the right hand columns in (B) and (C). (D and E) Structured illumination images of centrioles (Sas6, green; D-Plp, red) at the indicated cell cycle stages of control dsRNA-treated cells (upper panels), Ana2 dsRNA (D, lower panel), or Plk4 dsRNA (E, lower panel)-treated cells. Scale bars represent 0.5 μm.

With this knowledge, we could then address the interdependencies of Ana2 and Sas6 for their loading onto centrioles. We found that both Ana2-WT and the Ana2-4A mutant localized to centrioles, arguing that Ana2 recruitment does not require its Plk4-dependent association with Sas6 (Figure 3A). We then explored the ability of endogenous Ana2 to localize to centrioles in the absence of Sas6 and found this to be largely unaltered (Figure 3B). Thus Ana2 does not require Sas6 for its localization to centrioles. We then explored the reciprocal possibility by depleting Ana2 and assessing the localization of Sas6. We found that this diminished the level of Sas6 by 2- to 3-fold and resulted in centrioles that had only a single central punctum of Sas6. Sas6 failed to load onto anaphase/telophase centrioles in Ana2-depleted cells so that the majority of interphase centrioles retained only a single Sas6 punctum (Figures 3C and 3D). Thus Sas6 loading and the consequential formation of the procentriole are dependent on Ana2. Because phosphorylation of Ana2 by Plk4 is required for Ana2 to bind Sas6, we determined the effects of Plk4 depletion and found that this had similar consequences to *ana2* RNAi for Sas6 loading (Figures 3C and 3E). This accords with a requirement for Ana2 to be phosphorylated by Plk4 in order to interact with and therefore load Sas6 onto the centriole.

**Figure 4:**
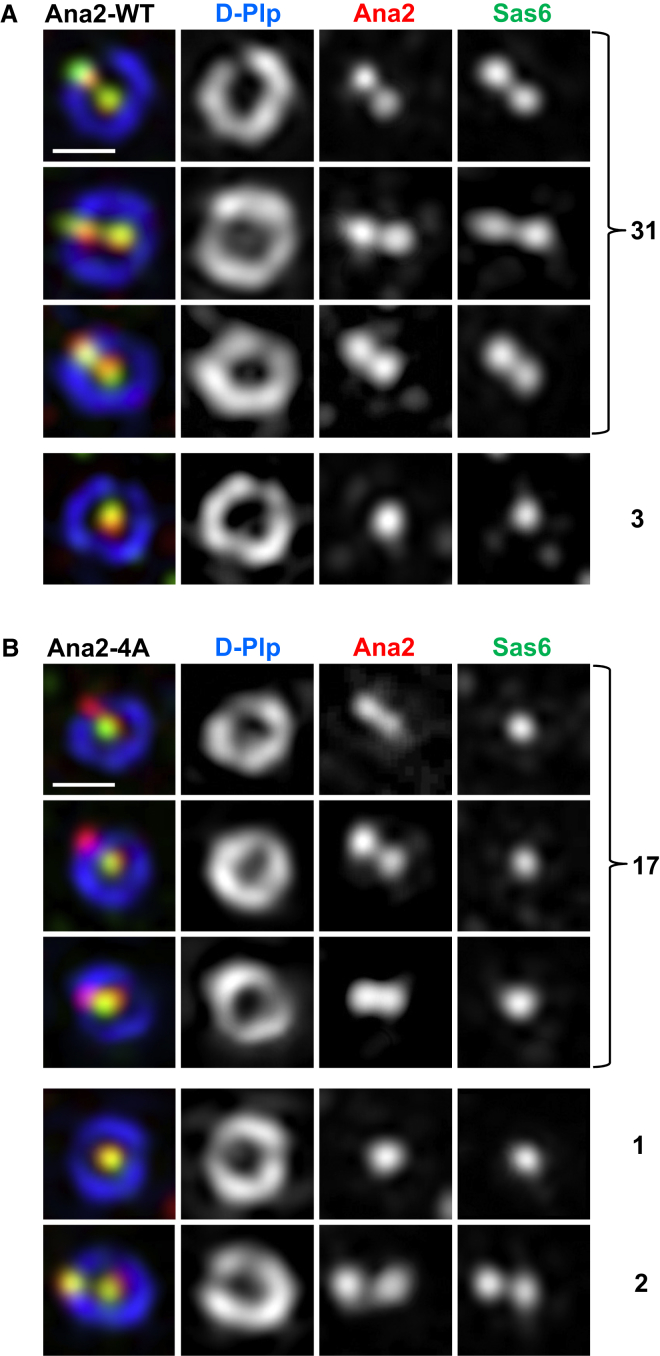
Plk4-Mediated Phosphorylation of Four Serine Residues within the STAN Motif of Ana2 Is Critical for Sas6 Recruitment to Centrioles Endogenous Ana2 was depleted using dsRNA directed against its UTRs in two cell lines, one stably expressing wild-type Ana2 (Ana2-WT) and the other, the alanine substitution mutant at the Plk4 sites in the STAN motif (Ana2-4A). Cells were immunostained to reveal the indicated proteins and analyzed by structured illumination microscopy. (A) The substituting Ana2-WT, both Ana2-WT and endogenous Sas6 colocalize at both central and the peripheral puncta in the great majority of interphase centrioles. (B) Ana2-4A loads onto mother and procentriole and Sas6 only onto the mother in the great majority of interphase centrioles. Scale bars represent 0.5 μm. Numbers of centrioles observed in each category are indicated on the right.

Finally, to determine whether phosphorylation of the four Plk4 sites within the STAN motif was critical for Sas6 recruitment, we asked whether Ana2-4A would block the recruitment of Sas6. For this purpose we depleted the endogenous Ana2 from our cell lines overexpressing either Ana2-WT or Ana2-4A and simultaneously monitored the localization of the transgenic Ana2 proteins and endogenous Sas6. The great majority (91%, n = 34) of interphase centrioles in cells with endogenous Ana2 substituted by Ana2-WT had two colocalizing puncta of Ana2 and Sas6 on both mother and daughter/procentriole (Figure 4) as expected from our above study of untreated cells (Figure S3). In striking contrast, when endogenous Ana2 was substituted with Ana2-4A, the majority (85%, n = 20) of interphase centrioles had puncta of Ana2 on mother and daughter, but Sas6 was associated only with the mother (Figure 4). This further demonstrates that Ana2 is able to load onto the site of procentriole formation irrespective of whether it can be phosphorylated by Plk4. However, Plk4-mediated phosphorylation of Ana2 is necessary in order to recruit Sas6 for procentriole formation.

Together our findings suggest a series of events that include disengagement of centrioles at the end of mitosis and the initiation of procentriole formation accompanied by re-engagement by loading Sas6. This would accord with the finding that centrioles of Sas6 mutant spermatocytes in *Drosophila* lose both their 9-fold symmetry and their engagement [34]; the former being consistent with Sas6’s role in establishing the cartwheel structure, the latter suggesting that Sas6 is also required to maintain the orthogonal link between mother and daughter. Here we observe that disengagement of the mother/daughter pair occurs immediately following the maturation of the daughter centriole, a process that we see through completion of a ring of D-Plp that encircles the daughter’s Ana2 and Sas6. This has similarities to the Plk1-dependent maturation and disengagement of the mother/daughter centriole pair of human cells as they pass through mitosis [37, 38]. Indeed, Polo is required for centriole separation in *Drosophila* [39]. Effectively, these processes constitute duplication licensing; they activate a site on the daughter centriole and clear Sas6 from the perimeter of the mother, allowing both mother and daughter to initiate procentriole formation. In accord with this notion, we see the recruitment of new Ana2 and Sas6 onto the mother and daughter only once they have disengaged. It is of interest to compare our findings on Sas6 recruitment in *Drosophila* to events in human cells where Sas6 is destroyed during G1 [33]. A recent study has shown that Sas6 is first transiently recruited to the lumen of the mother centriole in S phase before being repositioned to the site of procentriole formation, events that are dependent upon Stil (human Ana2) and Plk4 [40]. This contrasts to *Drosophila* where Sas6 is stable at the core of the centriole once it is incorporated, and only its initial incorporation into procentrioles appears to be dependent on Ana2 and Plk4.

Our evidence strongly suggests that the very first act of procentriole formation requires Ana2 to be phosphorylated by Plk4. A mutant form of Ana2 unable to be phosphorylated at the Plk4 sites permits neither Sas6 recruitment nor centriole duplication, and depletion of either Plk4 or Ana2 similarly prevents the spatio-temporal events of Sas6 loading. Thus, although we cannot exclude the possibility that other protein kinases can also phosphorylate Ana2 in vivo, it seems most probable that Ana2’s phosphorylation by Plk4 initiates centriole duplication because Plk4 is the only known protein kinase whose activity is sufficient for de novo centriole formation. The phosphorylation of Ana2 in its STAN motif enables it to recruit Sas6, presumably to form a new cartwheel structure and establish engagement of the new procentrioles to both old mother and daughter.

Many intriguing questions remain. How does Ana2 itself become recruited onto the site of procentriole formation and how is new Ana2 (and hence Sas6) restricted to this single site? What is the architecture of the Ana2-Sas6 complex? As we progress further in understanding how the centriole components are pieced together and how these events are controlled by reversible phosphorylation and regulated protein stability, the answers to these questions will surely emerge.

## Supplemental Information

Document S1. Figures S1–S3 and Supplemental Experimental Procedures

Document S2. Article plus Supplemental Information
