# Supplementary material for: Plk4 Phosphorylates Ana2 to Trigger Sas6 Recruitment and Procentriole Formation
Source: Curr Biol. 2014 Nov 3;24(21):2526–32. doi: 10.1016/j.cub.2014.08.061 (PMC4229625; doi:10.1016/j.cub.2014.08.061)
Supplement: Document S1. Figures S1–S3 and Supplemental Experimental Procedures [file mmc1.pdf]

Current Biology, Volume *24*

Supplemental Information

Plk4 Phosphorylates Ana2 to Trigger Sas6 Recruitment and Procentriole Formation

Nikola S. Dzhindzhev, George Tzolovsky, Zoltan Lipinszki, Sandra Schneider, Ramona Lattao, Jingyan Fu, Janusz Debski, Michal Dadlez, and David M. Glover

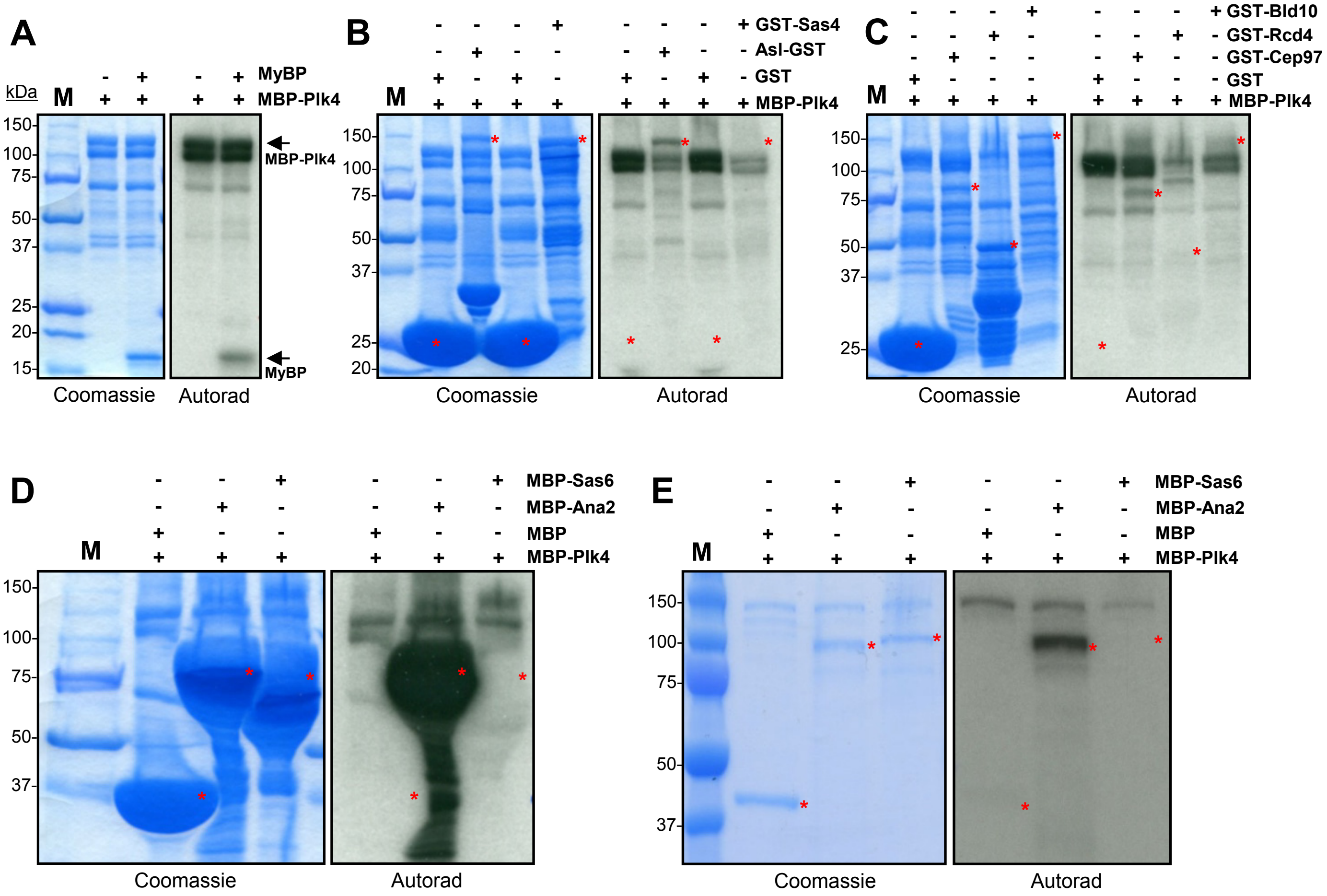

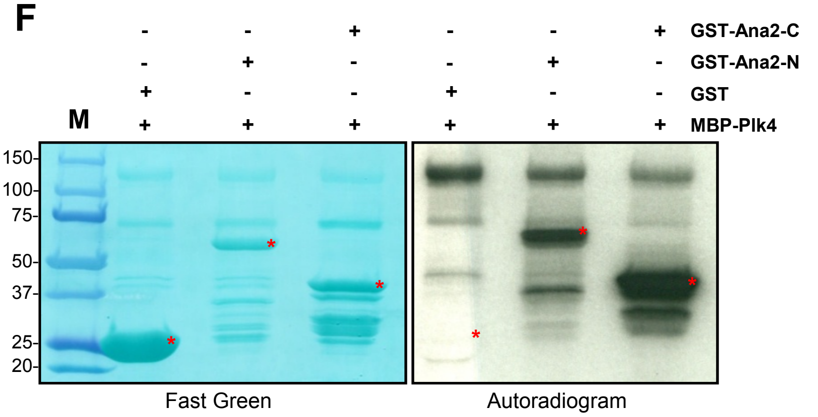

G

Position	*In vitro* Total number of spectral counts	*In vivo* Total number of spectral counts
T69	10	10
S84	13	70
T89	11	0
S98-105	2	15
S119	7	8
T124-125	16	0
S150-153	28	24
T159	62	0
T164	16	0
S177	23	16
S184	2	3
S234	1	3
S237-242	71	9
S318	120	1
S365-373	378	70

Figure S1

Figure S1 (Related to Figure 1): An *in vitro* kinase assay screen identifies Ana2 as a novel substrate of Plk4. (A) Recombinant and constitutively active Maltose Binding Protein-tagged Plk4^{T172E} (MBP-Plk4) was affinity purified and incubated with Myelin Basic Protein (MyBP) as artificial kinase substrate in the presence of ^{32}P-γ-ATP. Following SDS-PAGE and autoradiography it can be seen that MBP-Plk4 phosphorylates MyBP and also autophosphorylates itself demonstrating that the recombinant kinase is active *in vitro*. **(B-E)** Recombinant centriolar proteins tagged with glutathione S-transferase (GST) or MBP, as indicated above the panels, were affinity-purified on the appropriate resins and subsequently incubated with MBP-Plk4 in the presence of ^{32}P-γ-ATP. The reaction mixture was then boiled in Laemmli sample buffer and subjected to SDS-PAGE. Gels were stained with Coomassie Brilliant Blue (left panels), photographed, dried and analysed by autoradiography (right panels). Red asterisks denote the positions of bands corresponding to the full-length recombinant putative substrates. The autoradiograms demonstrate that MBP-Plk4 could phosphorylate Ana2 strongly (**D** and **E**); and Asl (**B**) and Cep97 (**C**) weakly; but could not modify Sas4 (**B**); Bld10 or Rcd4 (**C**); or Sas6 (**D** and **E**). The overloaded gel (**D**) illustrates the strength and specifically of the phosphorylation of Ana2 by MBP-Plk4 *in vitro* as opposed to MBP-alone or MBP-Sas6. Normal loading is shown on panel (**E**). **(F)** *In vitro* kinase assay demonstrating that MBP-Plk4 could phosphorylate both GST-tagged N- terminal part of Ana2 (GST-Ana2-N; residues 1-280 delineated in Figure 1B) as well as the C-terminal part (GST-Ana2-C; residues 281-420, Figure 1B). **(G)** Following phosphorylation by MBP-Plk4 *in vitro*, GST-Ana2 was subjected to phospho-peptide mapping by mass spectrometry. The table (column: *in vitro*) summarises the number of times a particular phospho-peptide was detected. Two tryptic peptides, one containing S318, the other containing S365, S370 and S373 were preferentially phosphorylated by MBP-Plk4 *in vitro*. Protein A-Ana2 was affinity purified from cultured cells also expressing Plk4ND and subjected to phospho-peptide mapping by mass spectrometry. The table (column: *in vivo*) summarises the number of times a particular phospho-peptide was detected. Tryptic peptides containing S318, S365, S370 and S373 within the STAN motif were phosphorylated both *in vitro* and *in vivo*. Several residues identified in our study as phosphorylated by Plk4 were also reported to be phosphorylated in *Drosophila* Kc 167 cells in a phospho-proteome analysis (PhosphoPep [S12]).

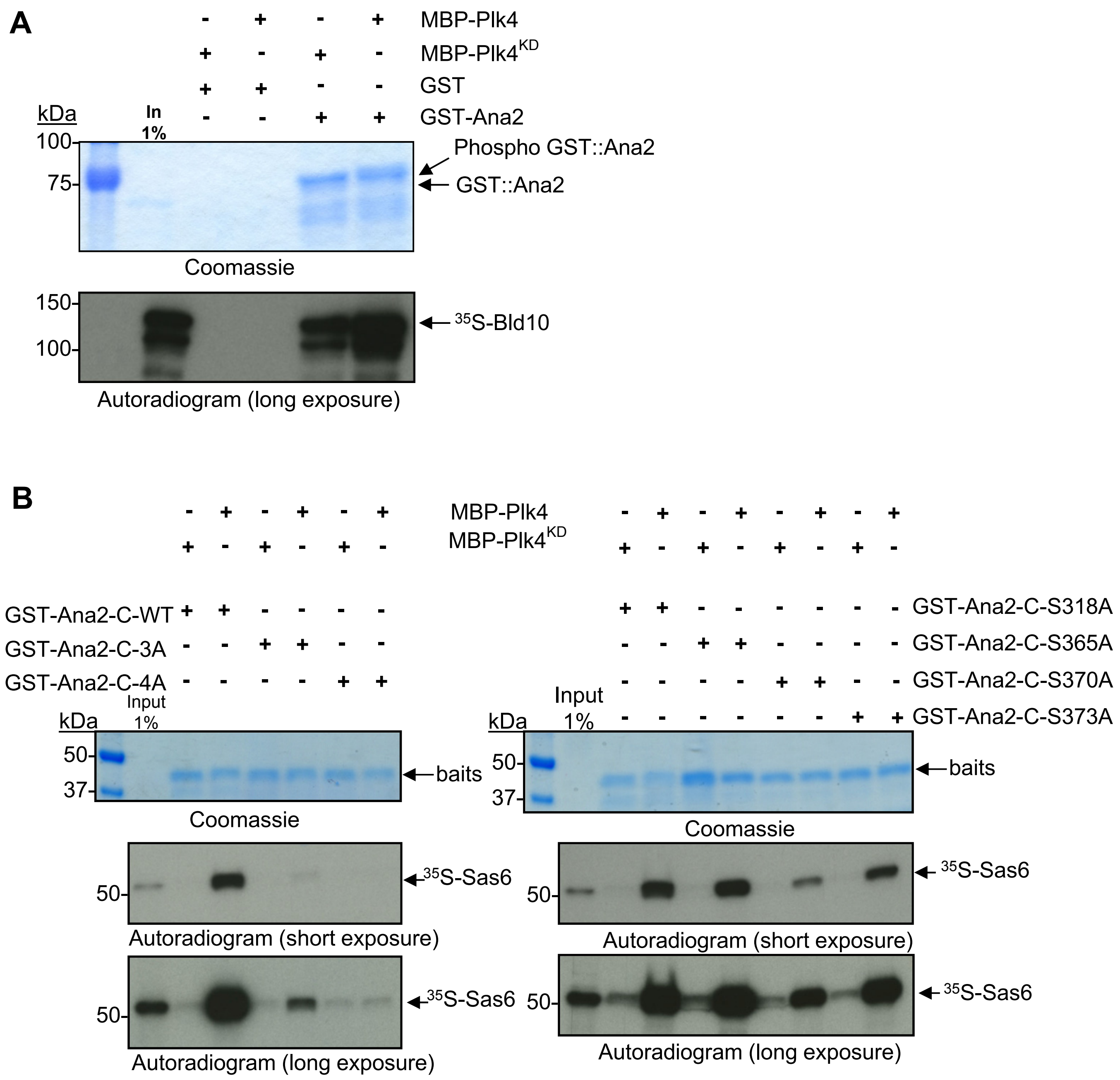
A
MBP-Plk4
MBP-Plk4KD
GST
GST-Ana2
kDa
In 1%
100-
75-
Phospho GST::Ana2
GST::Ana2
Coomassie
150-
100-
35S-Bld10
Autoradiogram (long exposure)
B
MBP-Plk4
MBP-Plk4KD
GST-Ana2-C-WT
GST-Ana2-C-3A
GST-Ana2-C-4A
Input 1%
kDa
50-
37-
baits
Coomassie
35S-Sas6
Autoradiogram (short exposure)
Autoradiogram (long exposure)
GST-Ana2-C-S318A
GST-Ana2-C-S365A
GST-Ana2-C-S370A
GST-Ana2-C-S373A

Figure S2

Figure S2 (Related to Figure 2): Plk4-mediated phosphorylation of four conserved serines within the STAN motif of Ana2 is essential for its interaction with Sas6. (A) A longer exposure of the autoradiogram shown in Figure 2A demonstrates that ^{35}S-Met-labelled Bld10 binds both non-phosphorylated and phosphorylated forms of Ana2, *in vitro*. **(B)** Mutation of all 4 conserved Ser-residues within the STAN-motif is required to completely abolish the Plk4-mediated Ana2-Sas6 interaction. ^{35}S-Met-labelled Sas6 was incubated with the following forms of the GST-tagged C-terminal part of Ana2: wild-type (GST-Ana2-C-WT) or with serine to alanine substitutions at residues 365, 370, 373 (GST-Ana2-C-3A), at residues 318, 365, 370, 373 (GST-Ana2-C-4A); or with single S-to-A point mutants at these sites (as indicated). The Ana2 variants were pre-treated with either active or inactive ($Plk4^{KD}$) MBP-tagged Plk4. Complexes were subjected to SDS-PAGE, gels were stained (Coomassie) and analysed by autoradiography. Among the individual serine to alanine substitutions, S370A results in the greatest reduction of Sas6 binding (right-hand panel, penultimate pair of lanes). However, all 4 conserved serine residues need to be mutated to completely disrupt the Ana2-Sas6 interaction (4A-mutant, left-hand panel, final pair of lanes).

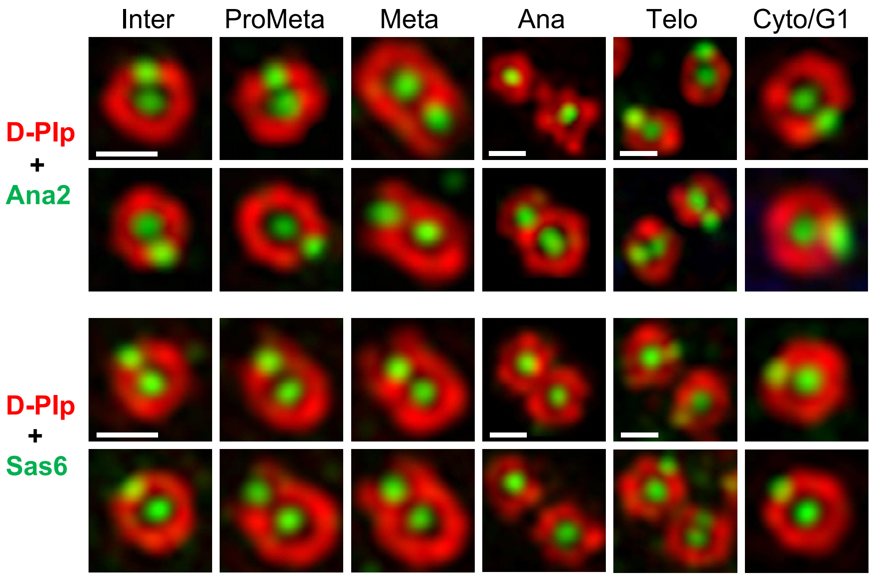

Figure S3

Figure S3 (Related to Figure 3): Centriolar localisation of endogenous Ana2 and Sas6 during the cell cycle. Localisation of endogenous Ana2 and Sas6 relative to D-Plp at the indicated cell cycle stages in D.Mel-2 cells revealed by structured illumination microscopy. At mitotic entry, Ana2 and Sas6 are both present in two discrete puncta, one in the centre of the mother centriole, the other marking the daughter that has yet to be surrounded by D-Plp. After completion of the D-Plp ring of the daughter centriole (metaphase/anaphase) and the disengagement of the mother- daughter pair in anaphase, both centrioles each acquire a new dot of Ana2 or Sas6 at their periphery during late-anaphase/telophase marking the site of the future procentriole formation. Scale bar, 0.5 μm. (Note: in anaphase and telophase the centrioles are shown at lower magnification; Scale bar, 0.5 μm).

Supplemental Experimental Procedures

DNA constructs

cDNA clones for Ana2 (clone LD22033), Sas6 [S1], Plk4 (clone RE70136), Sas4 [S2], Asl (clone GH02902), Cep97 (clone RE26466), Rcd4 (clone SD16838), Cep135/Bld10 (clone LD35990) and Ana1 (LD07765 and IP16240) were obtained from the *Drosophila* Genomics Resource Centre (DGRC), cloned in house or describes previously [S3]. Since no full-length clone for Ana1-RA was available, we created it by using two overlapping partial cDNA clones (LD07765 and IP16240). Full-length Ana2, Sas6, Plk4, Sas4, Asl, Cep97, Rcd4, Cep135/Bld10, Ana1, Ana2-N, Ana2-C and Sas6-C (residues 236-472) were respectively cloned into pDONR221 using the Gateway system (Life Technologies) according to the manufacturer's guidelines. Entry clones were verified by DNA sequencing. Plk4ND and Plk4^{NDKD} entry clones are described elsewhere [S4]. *Drosophila* expression vectors suitable for the *Actin5C* promoter-driven constitutive expression of chimeric proteins with eGFP-, 3xFLAG-, 6xMyc- or Protein A-tags fused to either the amino- or carboxy-terminus of the protein, respectively, were prepared using the following destination vectors obtained from DGRC or made in our lab [S5]: pAFW, pAWF, pAGW, pAWG, pAWM (*Drosophila* Gateway™ Vector Collection (https://emb.carnegiescience.edu/labs/murphy/Gateway%20vectors.html)), pAct5c-PrA (amino-terminal Protein A fusion under the regulation of Actin5c promoter) [S5] and pMT-cPrA (carboxy-terminal Protein A fusion under the regulation of the copper-inducible *Metallothionein A* (*CG9470*) promoter) [S5]. Plasmids for the expression of untagged Ana2-WT, Ana2-4A, Plk4ND and Plk4^{NDKD} were generated by recombining the STOP codon-containing entry clones into pAWF, respectively. Untagged Plk4ND was also made in pMT-cPrA using the same strategy. Due to the stop codon placed between the CDS and carboxy-terminal 3xFLAG- or Protein A-tags, the translated protein does not contain the tags (data not shown). Bacterial expression constructs for amino-terminal GST- or MBP-fusions were made using the following destination vectors: pDEST24 (Life Technologies) and pKM596 (Addgene plasmid 8837); [S6], respectively.

Cell culture, DNA and dsRNA transfections

Culturing of D.Mel-2 cells (Life Technologies) and RNAi techniques were described previously [S3, S5]. DNA transfections were carried out using the FuGENE-HD reagent according to the manufacturer's suggestion (Promega). Selection of stable cell lines was performed as described [S5, S7]. Oligonucleotide primers used to generate dsRNA are listed below or reported elsewhere [S1, S3]:

Ana2-F: 5'-GAATTAATACGACTCACTATAGGGAGAATGTTTGTTCCCGAAACGGAGG-3'
Ana2-R: 5'-GAATTAATACGACTCACTATAGGGAGACAGAGCCGCCAGATCACTCTTA-3'

For the efficient dsRNA-based silencing of endogenous Ana2, the following 225 bp-long hybrid DNA template (which contains the combination of *ana2*'s 5'-UTR and 3'-UTR) was generated by Overlap Extension PCR:

5'-GAATTAATACGACTCACTATAGGGAGACAGATTCTCCCGCTCGAATTTAATTTAATCGGCAAATATAAACAAAT ACGCTCCAAATGTTTGTTCCCGAACCGAAGCTGTTGTGAAAGCATGTACAATGTTCGTTTTGTTATTTATGCATAT GTCTATTTGCGATTTAAGTGGAAATATATTTCAATACACGGAAAAAAATCTCCCTATAGTGAGTCGTATTAATTC-3'

Site-directed mutagenesis

To create point mutants of Plk4 or Ana2, the QuikChange II XL Site-Directed Mutagenesis Kit was applied according to the manufacturer's guidelines (Agilent technologies) using wild-type cDNA or entry clones as template and the following oligonucleotide primers:

Plk4-T172E-F: 5'-AGCGACCTGATGAGCGCCATATGGAGATGTGTGGAACTCCGAACTATAT-3'
Plk4-T172E-R: 5'-ATATAGTTCGGAGTTCCACACATCTCCATATGGCGCTCATCAGGTCGCT-3'
Plk4-K43M-F: 5'-ACACTCACCAGGATGTGGCCATAATGATGATCGATAAAAAACTAATCCA-3'
Plk4-K43M-R: 5'-TGGATTAGTTTTTTATCGATCATCATTATGGCCACATCCTGGTGAGTGT-3'
Ana2-S318A-F: 5'-ACTGGCCAAGCCCAACACCGAGAAGGCAATGGTGATGAACGAGCTGGCGCTGA-3'
Ana2-S318A-R: 5'-TCAGCGCCAGCTCGTTCATCACCATTGCCTTCTCGGTGTTGGGCTTGGCCAGT-3'
Ana2-S365A-F: 5'-GATCGACAACATAGGCCACGCGCAGGCACCAAACGACATATCCAATGCTTCGT-3'
Ana2-S365A-R: 5'-ACGAAGCATTGGATATGTCGTTTGGTGCCTGCGCGTGGCCTATGTTGTCGATC-3'
Ana2-S370A-F: 5'-CCACGCGCAGAGTCCAAACGACATAGCAAATGCTTCGTACAAGTATCTCAAAA-3'
Ana2-S370A-R: 5'-TTTTGAGATACTTGTACGAAGCATTTGCTATGTCGTTTGGACTCTGCGCGTGG-3'
Ana2-S373A-F: 5'-GAGTCCAAACGACATATCCAATGCTGCATACAAGTATCTCAAAAAATACCGTC-3'
Ana2-S373A-R: 5'-GACGGTATTTTTTGAGATACTTGTATGCAGCATTGGATATGTCGTTTGGACTC-3'
Ana2-3A-F: 5'-CCACGCGCAGGCACCAAACGACATAGCAAATGCTGCATACAAGTATCTCAAAAAATACCGTC-3'
Ana2-3A-R: 5'-GACGGTATTTTTTGAGATACTTGTATGCAGCATTTGCTATGTCGTTTGGTGCCTGCGCGTGG-3'

Ana2-4A mutant was generated by using *Ana2-S318A-F and R* oligonucleotide primers and Ana2-3A entry clone as template.

Recombinant protein expression and purification

To obtain recombinant constitutively active Plk4 (MBP-Plk4), the activating T172E mutation was introduced into Plk4's CDS, while for the kinase-dead version (MBP-Plk4^{KD}), both T172E and the inactivating K43M mutations were introduced into the Plk4 sequence according to [S8]. The recombinant proteins were expressed in *E. coli* strain BL21 Star (DE3)pLysS (Life Technologies) following standard procedures and subsequently purified on Amylose resin (New England Biolabs) according to the manufacturer's instructions. The fusion proteins were eluted with Plk4 kinase buffer (Plk4-KB: 20 mM Na-HEPES pH 7.5, 100 mM NaCl, 10 mM $MgCl_2$, 10 mM $MnCl_2$, 1 mM DTT), supplemented with 15 mM D-Maltose. The quality of purified enzymes was analysed by SDS-PAGE. The concentration was estimated by spectrophotometry. Purified enzymes were stored at -80°C in Plk4-KB supplemented with 50% glycerol. MBP- and GST-tagged recombinant centrosomal proteins used in this study were expressed and purified as described previously [S3].

Protein purification from *Drosophila* cultured cells for phospho-residue mapping

For the affinity purification-coupled mass spectrometric analysis of Ana2 *in vivo* phosphorylation, stably transfected D.Mel-2 cells co-expressing Protein A-Ana2 (constitutive expression) and Plk4ND (expression induced by 0.5 mM $CuSO_4$ for 16 h) transgenic proteins were treated with 100 nM okadaic acid (OA, Calbiochem) for 2 h. Then, cells were harvested and whole cell lysate was prepared in extraction buffer [S9] supplemented with 100 nM OA and phosphatase inhibitor cocktail 2 and 3 (Sigma) as described previously [S5]. Detailed protocol for Protein A affinity purification and sample processing can be found in [S5, S9].

***In vitro* Plk4 kinase assay**

Typically, 4-10 μg of recombinant MBP-Plk4 or MBP-Plk4^{KD} were used in a 20-50 μl reaction volume for 30-60 minutes at 30 °C. For non-radioactive kinase assays substrates on beads were incubated with the enzyme in Plk4-KB supplemented with 250 μM ATP, while for radioactive kinase assay, the phosphorylation took place in the presence of 10 μM ATP and approximately 0.2 MBq of ^{32}P-γ-ATP.

Mass-spectrometry and phospho-peptide/residue mapping

Plk4-pre-phosphorylated recombinant GST-Ana2 or transgenic Protein A-Ana2 proteins were affinity-purified and digested with trypsin. Phosphorylated peptides were isolated using titanium dioxide, followed by LC/MS analysis as detailed in [S10].

***In vitro* binding assay**

Affinity-purified GST, GST-Ana2 (WT or 4A), GST-Ana2-N, GST-Ana2-C or point mutants of GST-Ana2-C were kept on Glutathione Sepharose 4B resin (GE Healthcare) and used as substrates for Plk4 and bait in binding assays. Bait proteins (~4-5 μg) were treated with MBP-Plk4 or MBP-Plk4^{KD} as explained above. Then the baits were supplemented with 600 μl binding buffer (50 mM Na-HEPES pH 7.5, 100 mM NaCl, 2 mM $MgCl_2$, 1 mM EGTA, 1 mM DTT, 0.1 % Triton-X 100, PhosStop (Roche), EDTA-free complete protease inhibitor cocktail (Roche), 100 nM okadaic acid (Calbiochem) and 0.5 mg/ml BSA) and incubated for 2 h at 4 °C with 15 μL ^{35}S-Met-labelled Sas6. ^{35}S-Sas6 was expressed in coupled *in vitro* transcription/translation reaction according to [S3]. After several washes with binding buffer (without BSA) beads were settled, boiled in 2x Laemmli sample buffer and subjected to SDS-PAGE followed by Coomassie Brilliant Blue staining. Gels were scanned, dried and used for autoradiography.

Co-immunoprecipitation

D.Mel-2 cells were transiently co-transfected with Actin5C-promoter-driven constructs encoding 3xFLAG-Ana2 (WT or 4A), Sas6-6xMyc or Plk4 (ND or NDKD) for 22 h. Cell were treated with 25 μM MG132 (Sigma) for 2.5 h, harvested and lysed in 1 ml ice-cold buffer containing 20 mM Tris–HCl pH 7.5, 150 mM NaCl, 2 mM $MgCl_2$,0.5 mM Na-EGTA pH 8.0, 1 mM DTT, 0.1 % NP-40, 5 % glycerol, 1 mM PMSF, EDTA-free complete protease inhibitor cocktail, Roche, supplemented with 100 nM okadaic acid and PhosStop (Roche) by 6x passing through a pre-chilled G25 needle. Lysates were clarified by centrifugation (4 °C, 10,000 g, 10 min) and supernatants were mixed with anti-FLAGM_2 magnetic beads (Sigma) for 3 h at 4 °C. After several washes proteins were eluted with 150 μg/ml 3xFLAG peptide, mixed with Laemmli sample buffer and subjected to SDS-PAGE followed by immunoblotting using standard procedures.

Antibodies

MBP-Ana2 (full length) and GST-Sas6-C (residues 236-472) were expressed and purified from *E. coli* and used as antigens for immunization of rats (Animal Facility at Instituto de Biologia Molecular e Celular, Porto, Portugal). GST-Ana2-N (residues 1-280) was used for production of a rabbit polyclonal antibody (Harlan Laboratories). GST-Plk4$^{301-600}$ and GST-Plk4$^{601-769}$ were expressed and purified from *E.coli*, combined and used to immunize sheep (Scottish National Blood Transfusion Service). Rabbit and sheep immunoglobulins were purified on Protein A or G sepharose according to the manufacturer's suggestion (GE Healthcare). We used the following primary antibodies for immuno-fluorescence (IF) or western blotting (WB): Rat-anti-Ana2 (this study; WB 1:4,000); Rabbit-anti-Asl [S3], WB 1:2,000; Sheep-anti-Plk4 (this study; WB 1:2,000); Rabbit-anti-Ana2 (this study; IF 1:1,000); Rat-anti-Sas6 (this study; IF 1:1,000); Chicken-anti-D-Plp [S2] IF 1:1,000; Mouse-anti-α-tubulin (clone DM1A Sigma, WB 1:5,000); Mouse-anti-FLAG (clone M2, Sigma, WB 1:20,000); Mouse-anti-Myc (clone 9E10, Abcam, WB 1:5,000).

Immunostaining and fluorescence microscopy

These procedures were described here [S3]. Briefly, D.Mel-2 cells were grown on concanavalinA-coated coverslips for 2–4h and fixed in cold methanol. Blocking (10% foetal calf serum), antibody incubations and washes were done in PBS, supplemented with 0.1% Triton X-100. Cells were mounted in Vectashield media with DAPI (Vector laboratories). Microscopic analysis was performed on a Carl Zeiss Axiovert 200M microscope with 40x/1; 63x/1.25 and 100x/1.4 Plan Apochromat objectives. Images were acquired with a Photometrics Cool SNAP HQ2 camera and the image analysis software Metamorph (v7.7).

Structured illumination microscopy:

Super-resolution images were acquired on an OMX-V3 system using a 63x/1.4NA oil Olympus lens as described previously [S11]. Images (512x512 ppi) were reconstructed and registered using the SoftWorx Linux package. Images were further processed to obtain maximum intensity projections. These were cropped and assembled in Photoshop v6.

Supplemental references

S1. Rodrigues-Martins, A., Bettencourt-Dias, M., Riparbelli, M., Ferreira, C., Ferreira, I., Callaini, G., and Glover, D.M. (2007). DSAS-6 organizes a tube-like centriole precursor, and its absence suggests modularity in centriole assembly. Current biology : CB *17*, 1465-1472.

S2. Rodrigues-Martins, A., Riparbelli, M., Callaini, G., Glover, D.M., and Bettencourt-Dias, M. (2007). Revisiting the role of the mother centriole in centriole biogenesis. Science *316*, 1046-1050.

S3. Dzhindzhev, N.S., Yu, Q.D., Weiskopf, K., Tzolovsky, G., Cunha-Ferreira, I., Riparbelli, M., Rodrigues- Martins, A., Bettencourt-Dias, M., Callaini, G., and Glover, D.M. (2010). Asterless is a scaffold for the onset of centriole assembly. Nature *467*, 714-718.

S4. Cunha-Ferreira, I., Rodrigues-Martins, A., Bento, I., Riparbelli, M., Zhang, W., Laue, E., Callaini, G., Glover, D.M., and Bettencourt-Dias, M. (2009). The SCF/Slimb ubiquitin ligase limits centrosome amplification through degradation of SAK/PLK4. Current biology : CB *19*, 43-49.

S5. D'Avino, P.P., Archambault, V., Przewloka, M.R., Zhang, W., Laue, E.D., and Glover, D.M. (2009). Isolation of protein complexes involved in mitosis and cytokinesis from Drosophila cultured cells. Methods in molecular biology (Clifton, N.J.) 545, 99-112.

S6. Fox, J.D., Routzahn, K.M., Bucher, M.H., and Waugh, D.S. (2003). Maltodextrin-binding proteins from diverse bacteria and archaea are potent solubility enhancers. FEBS Lett *537*, 53-57.

S7. D'Avino, P.P., Savoian, M.S., Capalbo, L., and Glover, D.M. (2006). RacGAP50C is sufficient to signal cleavage furrow formation during cytokinesis. J Cell Sci *119*, 4402-4408.

S8. Swallow, C.J., Ko, M.A., Siddiqui, N.U., Hudson, J.W., and Dennis, J.W. (2005). Sak/Plk4 and mitotic fidelity. Oncogene *24*, 306-312.

S9 Lipinszki, Z., Wang, P., Grant, R., Lindon, C., Dzhindzhev, N.S., D'Avino, P.P., Przewloka, M.R., Glover, D.M., and Archambault, V. (2014). Affinity purification of protein complexes from Drosophila embryos in cell cycle studies. Methods in molecular biology (Clifton, N.J.) 1170, 571-588.

S10 Graczyk, D., Debski, J., Muszynska, G., Bretner, M., Lefebvre, O., and Boguta, M. (2011). Casein kinase II-mediated phosphorylation of general repressor Maf1 triggers RNA polymerase III activation. Proceedings of the National Academy of Sciences of the United States of America *108*, 4926-4931.

S11. Fu, J., and Glover, D.M. (2012). Structured illumination of the interface between centriole and peri- centriolar material. Open biology *2*, 120104.

S12. Bodenmiller, B., Malmstrom, J., Gerrits, B., Campbell, D., Lam, H., Schmidt, A., Rinner, O., Mueller, L.N., Shannon, P.T., Pedrioli, P.G., et al. (2007). PhosphoPep--a phosphoproteome resource for systems biology research in Drosophila Kc167 cells. Molecular systems biology *3*, 139.
