# Supplementary material for: Plk4 Phosphorylates Ana2 to Trigger Sas6 Recruitment and Procentriole Formation
Source: Curr Biol. 2014 Nov 3;24(21):2526–32. doi: 10.1016/j.cub.2014.08.061 (PMC4229625; doi:10.1016/j.cub.2014.08.061)
Supplement: Document S2. Article plus Supplemental Information [file mmc2.pdf]

Current Biology 24, 2526–2532, November 3, 2014 ©2014 The Authors http://dx.doi.org/10.1016/j.cub.2014.08.061

Report

Plk4 Phosphorylates Ana2 to Trigger Sas6 Recruitment and Procentriole Formation

Nikola S. Dzhindzhev,[1,*] George Tzolovsky,[1] Zoltan Lipinszki,[1] Sandra Schneider,[1] Ramona Lattao,[1] Jingyan Fu,[1] Janusz Debski,[2] Michal Dadlez,[2] and David M. Glover[1,*]
[1]Department of Genetics, University of Cambridge, Cambridge CB2 3EH, UK
[2]Laboratory of Mass Spectrometry, Institute of Biochemistry and Biophysics, Polish Academy of Sciences, 02-106 Warsaw, Poland

Summary

Centrioles are 9-fold symmetrical structures at the core of centrosomes and base of cilia whose dysfunction has been linked to a wide range of inherited diseases and cancer [1]. Their duplication is regulated by a protein kinase of conserved structure, the *C. elegans* ZYG-1 or its Polo-like kinase 4 (Plk4) counterpart in other organisms [2–4]. Although Plk4's centriolar partners and mechanisms that regulate its stability are known, its crucial substrates for centriole duplication have never been identified. Here we show that *Drosophila* Plk4 phosphorylates four conserved serines in the STAN motif of the core centriole protein Ana2 to enable it to bind and recruit its Sas6 partner. Ana2 and Sas6 normally load onto both mother and daughter centrioles immediately after their disengagement toward the end of mitosis to seed procentriole formation. Nonphosphorylatable Ana2 still localizes to the centriole but can no longer recruit Sas6 and centriole duplication fails. Thus, following centriole disengagement, recruitment of Ana2 and its phosphorylation by Plk4 are the earliest known events in centriole duplication to recruit Sas6 and thereby establish the architecture of the new procentriole engaged with its parent.

Results and Discussion

We now have quite detailed knowledge of the partners of the Plk4 family of kinases at centrioles. In *C. elegans,* ZYG-1 is targeted to centrioles by SPD-2 [5, 6] whereas in *Drosophila*, Asterless has this function [7]. Targeting in mammalian cells requires the respective counterparts of both proteins Cep192 and Cep152 [8, 9] that can each interact with Plk4's cryptic polo-box domain. Procentriole formation can be initiated at multiple sites not only when Plk4 is overexpressed [3, 4, 10] or when its SCF-dependent proteolysis is prevented [11, 12], but also when expression of its targeting subunit is elevated [7]. Despite this extensive knowledge, the identity of Plk4's critical substrate for centriole duplication has remained elusive. Several substrates of Plk4/ZYG-1 have been identified to date that include SAS-6 [13], Cep152 [14], and a component of γ-TuRC, Gcp6 [15], but it is not clear how phosphorylation of these proteins might affect centriole duplication. To address this question we chose to identify centriole proteins that could be phosphorylated by Plk4 in vitro. To this end we purified an active form of *Drosophila* Plk4 expressed in *E. coli* that was able to undertake known autophosphorylation [16–20] and was also active toward an artificial substrate (Figure S1A available online). We first tested whether this preparation of Plk4 would phosphorylate proteins found in the outer layers of the centriole [21, 22]. This revealed that Plk4 could weakly phosphorylate its partner protein, Asl (Figure S1B), and Cep97 (Figure S1C), a protein that complexes with the Cp110 centriole capping protein [23]. However, it could not phosphorylate the microtubule wall-associated protein, Sas4 [21, 24–26], (Figure S1B); Rcd4, a poorly characterized centriole duplication protein (Figure S1C); or Bld10/Cep135, a protein required for maintenance but not formation of the core centriole [27, 28] (Figure S1C). We then asked whether the core centriole proteins Ana2 and Sas6 might be substrates as both are essential for centriole duplication in *Drosophila* [29, 30] and their respective counterparts in *C. elegans*, SAS-5 and SAS-6, are immediately downstream of ZYG-1 in the recruitment hierarchy of centriole proteins in *C. elegans* [5, 6]. Strikingly, Plk4 could strongly phosphorylate Ana2 but not Sas6 (Figures 1A, S1D, and S1E), suggesting the possibility that Ana2 might be the Plk4 substrate that triggers centriole duplication.

To test the above hypothesis, we first mapped the sites on Ana2 phosphorylated by Plk4 in vitro and tested the significance of their modification. Mass spectrometric analysis revealed multiple Plk4 phosphorylation sites of which four serine residues (S318, S365, S370, and S373) (Figures 1B and 1C, arrows, Figure S1G) stood out because their total spectral counts (times a particular phospho-peptide was detected) were much higher than any others. Moreover, they seemed to be the only Plk4 sites in the C-terminal part of Ana2 as their mutation to alanine prevented phosphorylation by Plk4 in vitro (Figures 1D and S1F). Their functional importance was also suggested by their conservation within the STAN motif that characterizes Ana2 orthologs (Figure 1C) and the finding that phosphorylation of the same sites could be detected in vivo (see Figure S1G and legend). To test their biological significance, we asked whether Ana2 with alanine substitutions at these sites (Ana2-4A) would permit centriole duplication. For this purpose we first established two D.Mel-2 cell lines, stably expressing untagged versions of either wild-type Ana2 (Ana2-WT) or Ana2-4A that each lacked the UTRs of the endogenous gene. We then depleted endogenous Ana2 from these lines using dsRNA against its UTRs. Three 4-day treatments of control D.Mel-2 cells with ds *ana2-UTR* RNA led to complete loss of centrioles from 68% of cells (data not shown). By contrast, depletion of endogenous Ana2 from the line expressing the Ana2-WT transgene had no significant effect upon centriole number, indicating that it can fully substitute for the endogenous protein. However, expression of transgenic Ana2-4A not only failed to rescue endogenous Ana2 depletion, but also had a significant dominant-negative effect, an increased proportion of cells lacking centrioles following control-RNAi (Figures 1E and 1F). Together this demonstrates the functional importance of these four conserved serines in the STAN motif of Ana2 for centriole duplication.

We next considered whether phosphorylation of Ana2 by Plk4 might affect its interaction with other components of the

*Correspondence: nsd23@cam.ac.uk (N.S.D.), dmg25@cam.ac.uk (D.M.G.)
This is an open access article under the CC BY license (http://creativecommons.org/licenses/by/3.0/).

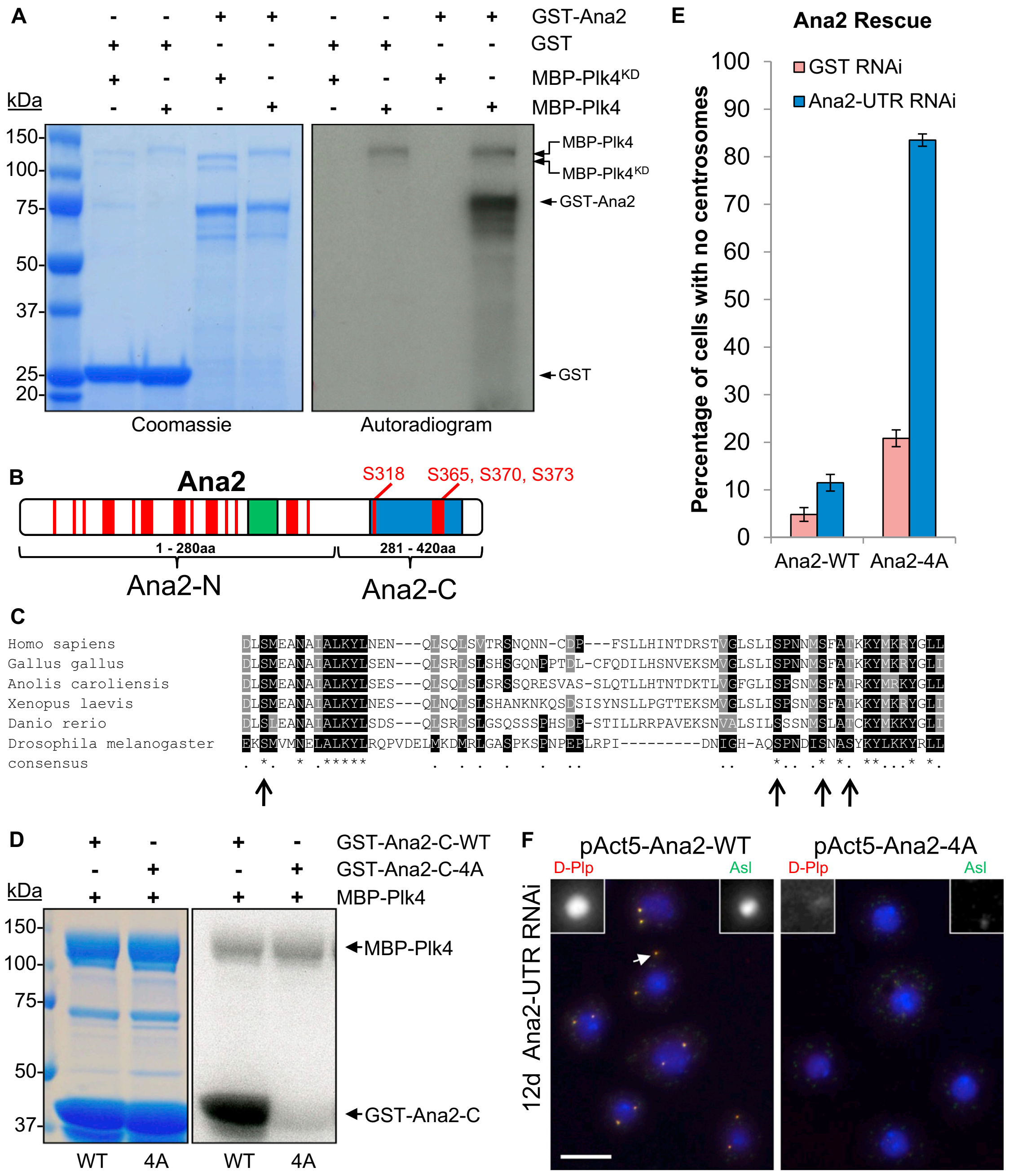

Figure 1. Plk4 Phosphorylates Ana2 at Four Essential Serine Residues in the Conserved STAN Motif

(A) GST-Ana2 or GST were incubated with active (MBP-Plk4) or inactive (MBP-Plk4^{KD}) Plk4 in the presence of γ-[^{32}P]ATP and run on SDS-PAGE (Coomassie), photographed, dried, and directly subjected to autoradiography.

(B) Plk4 phosphorylation sites (red bars) revealed by mass spectrometry (see Figure S1G) of which four serines are in the conserved STAN motif (blue box). Green box is predicted coiled-coil.

(C) Alignment of STAN motifs in Ana2 orthologs showing the four conserved Plk4 phosphorylation sites (arrows).

(D) Plk4 phosphorylates C-terminal portion (indicated in B) of wild-type Ana2 (GST-Ana2-C-WT) but not mutant with alanine substitutions at the four mapped Plk4 sites (GST-Ana2-C-4A).

(E) Cell lines overexpressing either Ana2-WT or Ana2-4A from the *actin5c* promoter were treated with either control dsRNA (GST-RNAi) or dsRNA targeting the UTRs of endogenous Ana2 (Ana2-UTR RNAi). Cells with no centrosomes were scored after three rounds (12 days in total) of dsRNA treatment. Error bars represent SEM.

(F) Micrographs from (E) showing centrosomes revealed by D-Plp and Asl costaining in the two cell lines following Ana2-UTR RNAi. Insets show D-Plp and Asl in monochrome from one pole (indicated by a white arrow). DNA is stained with DAPI (blue). Scale bar represents 10 μm.

centriole duplication machinery. To this end we loaded recombinant GST-Ana2 onto beads, incubated with either active or inactive (Plk4^{KD}) kinase and then with ^{35}S-methionine-labeled centriole proteins synthesized in vitro. We were unable to detect binding of Ana2 to either Rcd4 or Ana1; its binding to Sas4 showed no change; and its weaker binding to Bld10

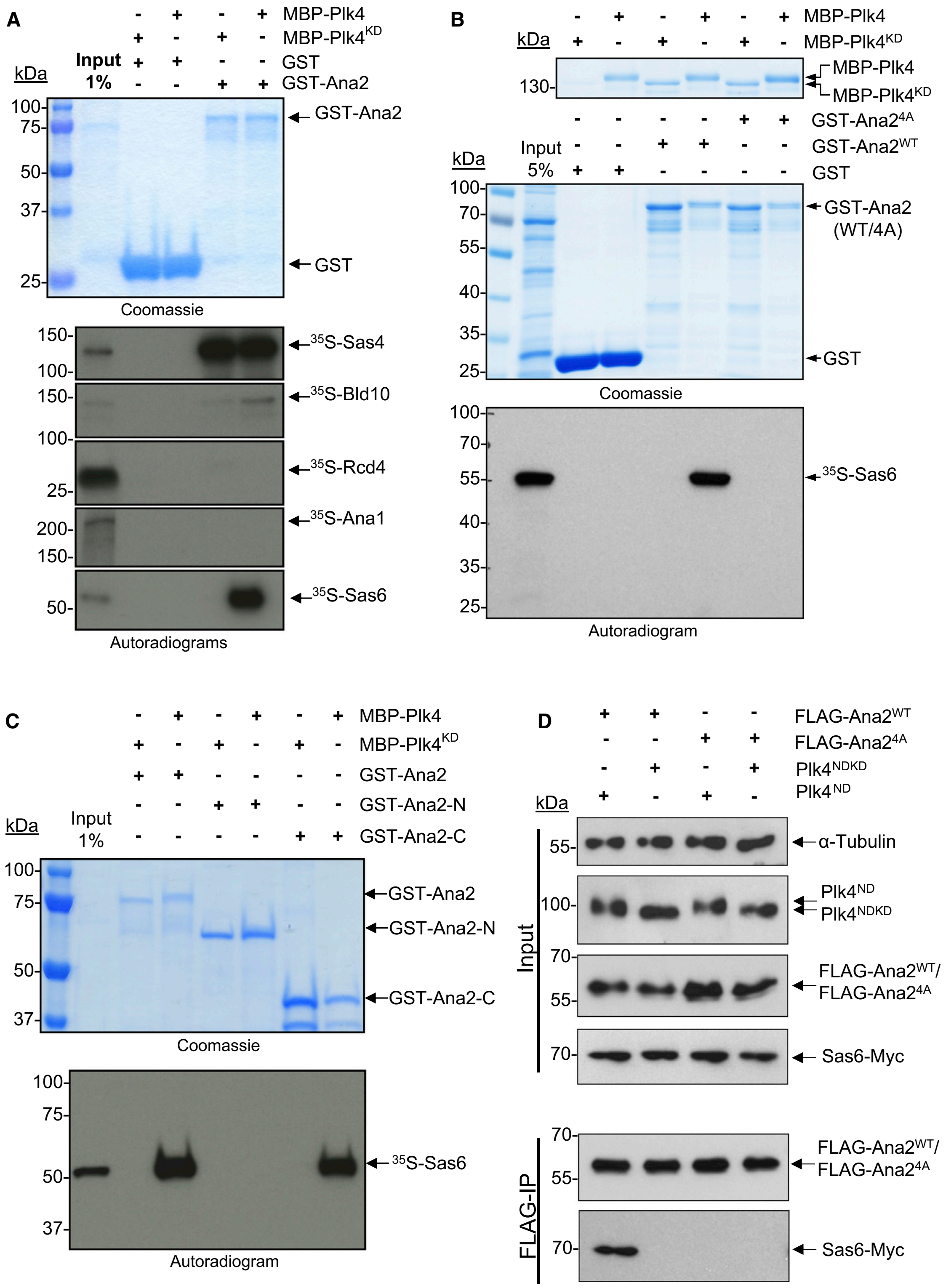

Figure 2. Plk4 Triggers a Direct, Phosphorylation-Dependent Interaction between Ana2 and Sas6 In Vitro and In Vivo

(A) In vitro screen for differential interactions of centriolar proteins with Ana2 with and without phosphorylation by Plk4. GST-Ana2 treated with active (MBP-Plk4) or inactive (MBP-Plk4^{KD}) Plk4 and incubated with ^{35}S-Met-labeled Sas4, Bld10, Rcd4, Ana1, or Sas6 synthesized by in vitro transcription and translation. Affinity-purified complexes were analyzed by SDS-PAGE (Coomassie) followed by autoradiography. Note: Sas6 interacts with Plk4-prephosphorylated Ana2 but not with Ana2 treated with the inactive kinase. See Figure S2A for longer exposure showing interaction with Bld10.

(B) GST, GST-tagged wild-type Ana2 (GST-Ana2-WT), or the four alanine substitution mutant (GST-Ana2-4A) were treated with either MBP-Plk4 or MBP-Plk4^{KD} and incubated in vitro with ^{35}S-Met-labeled Sas6. The resulting complex was analyzed by SDS-PAGE (Coomassie) and autoradiography. Sas6 specifically interacts with Ana2, when the four conserved serines in the STAN motif are pre-phosphorylated by Plk4.

(legend continued on next page)

showed a 3.5-fold increase in response to Ana2's phosphorylation state (Figures 2A and S2A). However, Sas6 showed a dramatic increase in binding to Ana2 phosphorylated by Plk4 (Figures 2A and 2B). The C-terminal part of Ana2 containing the STAN motif was necessary and sufficient for this phospho-dependent interaction with Sas6 (Figure 2C), leading us to test the consequences of mutations at its four Plk4 sites. We found that the Ana2-4A mutant was unable to interact with Sas6 even after incubation with active Plk4 (Figure 2B), indicating that phosphorylation on these sites is required. When we mutated individual serines to alanines, the strength of the interaction was diminished, particularly with S370A mutant, but not completely abolished (Figure S2B). Thus Plk4 phosphorylation of Ana2 on all four residues is critical to mediate its interaction with Sas6 in vitro. To validate these findings in vivo, we cotransfected D.Mel-2 cells with Sas6-Myc and FLAG-Ana2 (either WT or 4A) and either active Plk4 mutated in its degron ($Plk4^{ND}$) or inactive Plk4 ("nondegradable and kinase-dead" $Plk4^{NDKD}$). Following FLAG-pulldown we could detect Sas6 associated with Ana2-WT but not Ana2-4A and only when coexpressed with the active form of Plk4 (Figure 2D). This verifies our in vitro findings that following phosphorylation by Plk4, Ana2 is able to interact with Sas6.

Sas6 provides a structural basis for centriole architecture; its oligomers adopt a 9-fold symmetrical arrangement to form the cartwheel structure of the procentriole [31, 32]. Stil (human Ana2) and hSas6 are the first proteins to follow Plk4 to a dot-like structure marking assembly of the procentriole [9, 22, 33]. Sas6 is essential for correct centriole structure in *Drosophila* although, unlike Plk4, its overexpression does not lead to proper centriole formation in eggs [34]. However, boosting expression of both Sas6 and Ana2 stimulates formation of multiple microtubule organizing centers in eggs [35] and tubular aggregates linked to disengaged centrioles in spermatocytes [36]. Interestingly, however, such Sas6 and Ana2 could be recruited to centrioles only if Plk4 were also overexpressed in spermatocytes leading to centriole overduplication [36]. These earlier findings might be accounted for if the phosphorylation of Ana2 by Plk4 triggered the first step in cartwheel formation by Sas6, leading to procentriole formation.

To address the above hypothesis, we first needed to examine the progressive recruitment of Ana2 and Sas6 to centrioles in their duplication cycle relative to the outer centriolar marker D-Plp. At mitotic entry, each centrosome comprises a pair of orthogonally *engaged* centrioles, which we refer to as mother and daughter, surrounded by peri-centriolar, microtubule-nucleating material. The daughter centriole is immature at this stage and matures during mitosis [37]. In *Drosophila* cells, the mother centriole is encircled by a D-Plp ring and during maturation, two "horns" of D-Plp progressively extend around the daughter to give a complete ring by metaphase/early anaphase. Once the D-Plp ring is complete, the paired centrioles *disengage* during late anaphase so that each newly born cell exits cytokinesis into G1 with two well-separated centrioles (Figure S3). At mitotic entry, Sas6 and Ana2 are both present in two discrete puncta, one in the center of the mother centriole, the other marking the daughter and yet to become encircled by D-Plp. When the mother and mature daughter disengage, they each have a single dot of Ana2 or Sas6 at their center. Then, in late anaphase/telophase, a second Ana2/Sas6 dot appears at the periphery of each physically separated centrioles, marking the site of procentriole formation (Figure S3).

With this knowledge, we could then address the interdependencies of Ana2 and Sas6 for their loading onto centrioles. We found that both Ana2-WT and the Ana2-4A mutant localized to centrioles, arguing that Ana2 recruitment does not require its Plk4-dependent association with Sas6 (Figure 3A). We then explored the ability of endogenous Ana2 to localize to centrioles in the absence of Sas6 and found this to be largely unaltered (Figure 3B). Thus Ana2 does not require Sas6 for its localization to centrioles. We then explored the reciprocal possibility by depleting Ana2 and assessing the localization of Sas6. We found that this diminished the level of Sas6 by 2- to 3-fold and resulted in centrioles that had only a single central punctum of Sas6. Sas6 failed to load onto anaphase/telophase centrioles in Ana2-depleted cells so that the majority of interphase centrioles retained only a single Sas6 punctum (Figures 3C and 3D). Thus Sas6 loading and the consequential formation of the procentriole are dependent on Ana2. Because phosphorylation of Ana2 by Plk4 is required for Ana2 to bind Sas6, we determined the effects of Plk4 depletion and found that this had similar consequences to *ana2* RNAi for Sas6 loading (Figures 3C and 3E). This accords with a requirement for Ana2 to be phosphorylated by Plk4 in order to interact with and therefore load Sas6 onto the centriole.

Finally, to determine whether phosphorylation of the four Plk4 sites within the STAN motif was critical for Sas6 recruitment, we asked whether Ana2-4A would block the recruitment of Sas6. For this purpose we depleted the endogenous Ana2 from our cell lines overexpressing either Ana2-WT or Ana2-4A and simultaneously monitored the localization of the transgenic Ana2 proteins and endogenous Sas6. The great majority (91%, n = 34) of interphase centrioles in cells with endogenous Ana2 substituted by Ana2-WT had two colocalizing puncta of Ana2 and Sas6 on both mother and daughter/procentriole (Figure 4) as expected from our above study of untreated cells (Figure S3). In striking contrast, when endogenous Ana2 was substituted with Ana2-4A, the majority (85%, n = 20) of interphase centrioles had puncta of Ana2 on mother and daughter, but Sas6 was associated only with the mother (Figure 4). This further demonstrates that Ana2 is able to load onto the site of procentriole formation irrespective of whether it can be phosphorylated by Plk4. However, Plk4-mediated phosphorylation of Ana2 is necessary in order to recruit Sas6 for procentriole formation.

Together our findings suggest a series of events that include disengagement of centrioles at the end of mitosis and the initiation of procentriole formation accompanied by re-engagement by loading Sas6. This would accord with the finding that centrioles of Sas6 mutant spermatocytes in *Drosophila* lose both their 9-fold symmetry and their engagement [34]; the former being consistent with Sas6's role in establishing the cartwheel structure, the latter suggesting that Sas6 is also required to maintain the orthogonal link between mother and daughter. Here we observe that disengagement of the mother/daughter pair occurs immediately following the

(C) GST-Ana2, GST-Ana2-N (residues 1–280), or GST-Ana2-C (residues 281–420) incubated with either MBP-Plk4 or MBP-$Plk4^{KD}$ and ^{35}S-Met-labeled Sas6 and subjected to SDS-PAGE and autoradiography. C-terminal but not N-terminal part of Ana2 binds to Sas6 in vitro.

(D) FLAG-tagged wild-type Ana2 (FLAG-$Ana2^{WT}$) or the four alanine substitution mutant (FLAG-$Ana2^{4A}$) were transiently cooverexpressed with Myc-tagged Sas6 (Sas6-Myc) and either the degron mutant ($Plk4^{ND}$) or degron/kinase dead ($Plk4^{NDKD}$) forms of Plk4 in D.Mel-2 cells. Input and anti-FLAG-immunoprecipitates were subjected to SDS-PAGE and Western blotting to reveal the indicated antigens.

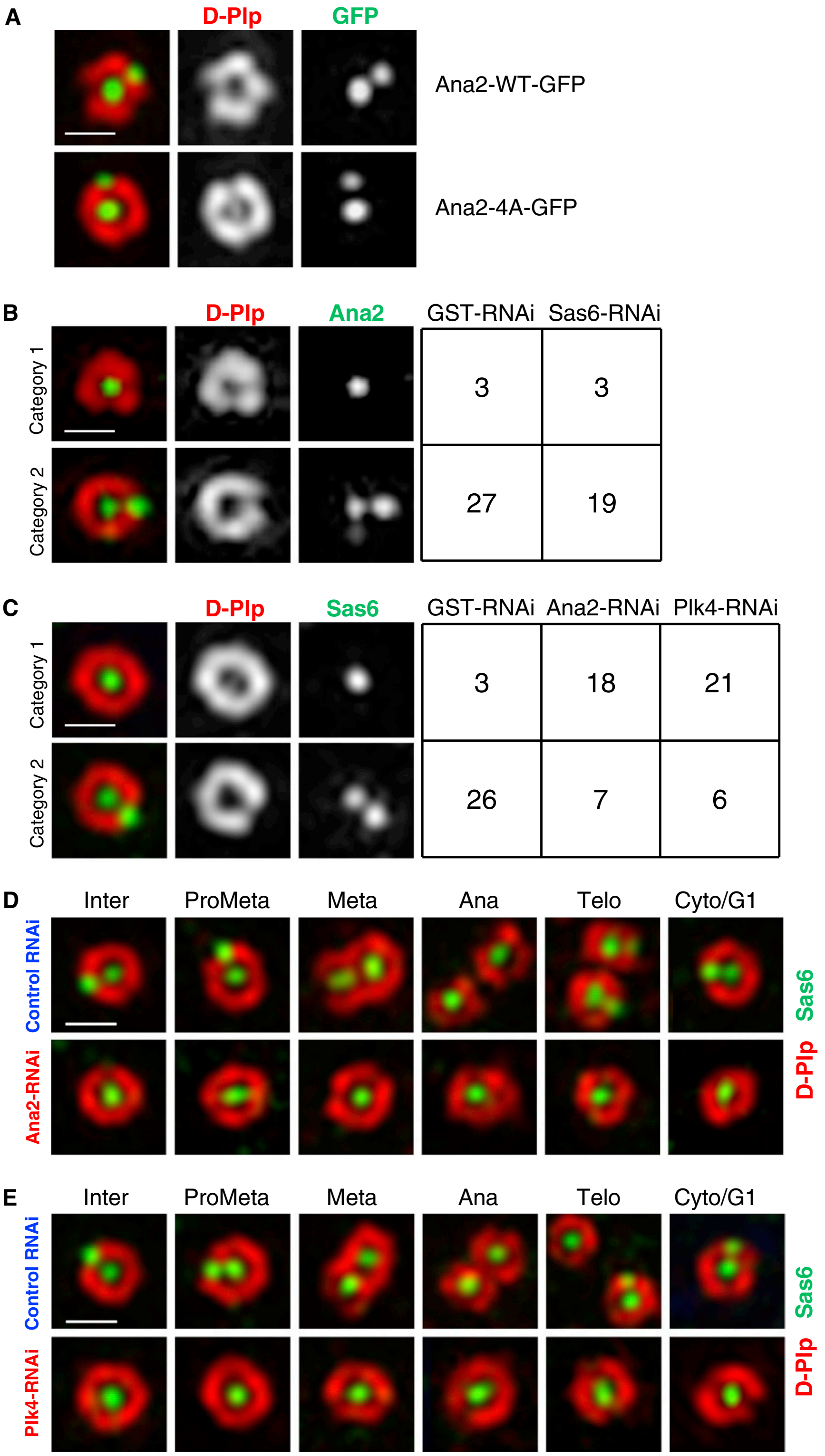

Figure 3. Plk4 and Ana2 Are Essential for Sas6 Loading onto Centrioles, while Ana2 Loading Is Independent of Sas6

(A) Structured illumination images of centrioles showing exogenous GFP-tagged wild-type Ana2 and its mutant form with alanine substitutions in the four serines phosphorylated by Plk4 (Ana2-WT-GFP and Ana2-4A-GFP, respectively) both associate with two distinct puncta in interphase centrioles (compare to endogenous Ana2; Figure S3). Note that endogenous Ana2 was not depleted in these experiments.

(B) Categories of Ana2 localization in interphase centrioles following control (GST dsRNA) or Sas6 RNAi.

(C) Categories of Sas6 localization following control (GST), Ana2, or Plk4 RNAi.

Numbers of centrioles counted and imaged are shown on the right hand columns in (B) and (C).

(D and E) Structured illumination images of centrioles (Sas6, green; D-Plp, red) at the indicated cell cycle stages of control dsRNA-treated cells (upper panels), Ana2 dsRNA (D, lower panel), or Plk4 dsRNA (E, lower panel)-treated cells.

Scale bars represent 0.5 μm.

maturation of the daughter centriole, a process that we see through completion of a ring of D-Plp that encircles the daughter's Ana2 and Sas6. This has similarities to the Plk1-dependent maturation and disengagement of the mother/daughter centriole pair of human cells as they pass through mitosis [37, 38]. Indeed, Polo is required for centriole separation in *Drosophila* [39]. Effectively, these processes constitute duplication licensing; they activate a site on the daughter centriole and clear Sas6 from the perimeter of the mother, allowing both mother and daughter to initiate procentriole formation. In accord with this notion, we see the recruitment of new Ana2 and Sas6 onto the mother and daughter only once they have disengaged. It is of interest to compare our findings on Sas6 recruitment in *Drosophila* to events in human cells where Sas6 is destroyed during G1 [33]. A recent study has shown that Sas6 is first transiently recruited to the lumen of the mother centriole in S phase before being repositioned to the site of procentriole formation, events that are dependent upon Stil (human Ana2) and Plk4 [40]. This contrasts to *Drosophila* where Sas6 is stable at the core of the centriole once it is incorporated, and only its initial incorporation into procentrioles appears to be dependent on Ana2 and Plk4.

Our evidence strongly suggests that the very first act of procentriole formation requires Ana2 to be phosphorylated by Plk4. A mutant form of Ana2 unable to be phosphorylated at the Plk4 sites permits neither Sas6 recruitment nor centriole duplication, and depletion of either Plk4 or Ana2 similarly prevents the spatio-temporal events of Sas6 loading. Thus, although we cannot exclude the possibility that other protein kinases can also phosphorylate Ana2 in vivo, it seems most probable that Ana2's phosphorylation by Plk4 initiates centriole duplication because Plk4 is the only known protein kinase whose activity is sufficient for de novo centriole formation. The phosphorylation of Ana2 in its STAN motif enables it to recruit Sas6,

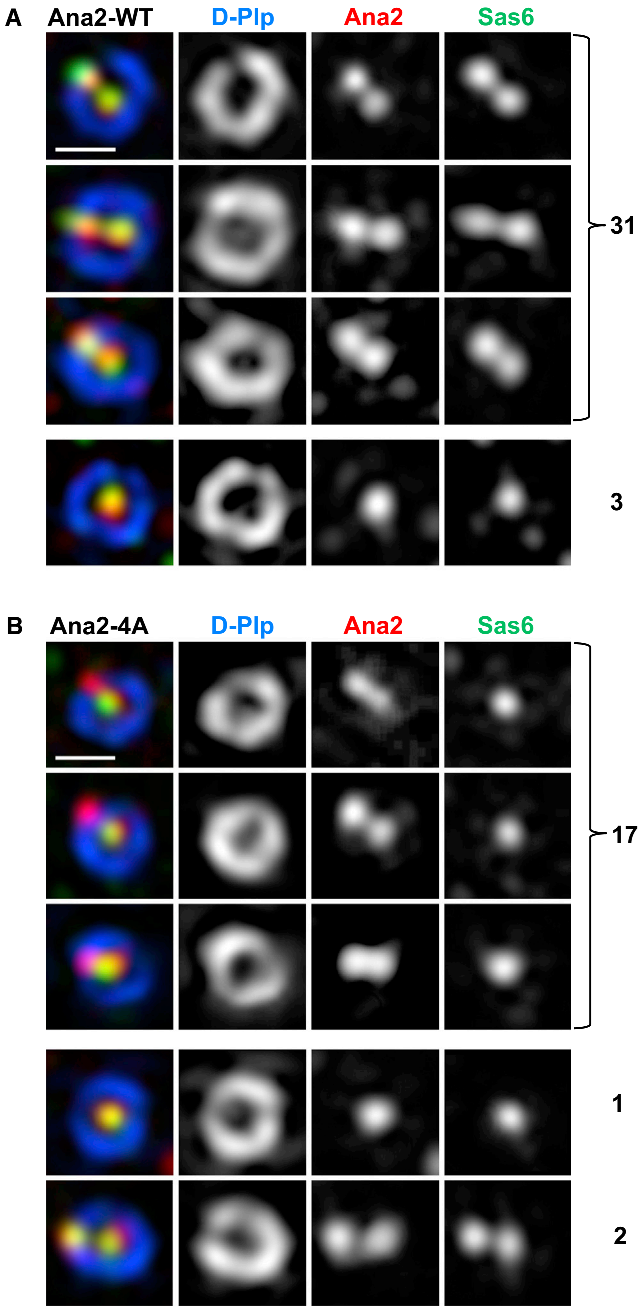

Figure 4. Plk4-Mediated Phosphorylation of Four Serine Residues within the STAN Motif of Ana2 Is Critical for Sas6 Recruitment to Centrioles

Endogenous Ana2 was depleted using dsRNA directed against its UTRs in two cell lines, one stably expressing wild-type Ana2 (Ana2-WT) and the other, the alanine substitution mutant at the Plk4 sites in the STAN motif (Ana2-4A). Cells were immunostained to reveal the indicated proteins and analyzed by structured illumination microscopy.

(A) The substituting Ana2-WT, both Ana2-WT and endogenous Sas6 colocalize at both central and the peripheral puncta in the great majority of interphase centrioles.

(B) Ana2-4A loads onto mother and procentriole and Sas6 only onto the mother in the great majority of interphase centrioles.

Scale bars represent 0.5 μm. Numbers of centrioles observed in each category are indicated on the right.

presumably to form a new cartwheel structure and establish engagement of the new procentrioles to both old mother and daughter.

Many intriguing questions remain. How does Ana2 itself become recruited onto the site of procentriole formation and how is new Ana2 (and hence Sas6) restricted to this single site? What is the architecture of the Ana2-Sas6 complex? As we progress further in understanding how the centriole components are pieced together and how these events are controlled by reversible phosphorylation and regulated protein stability, the answers to these questions will surely emerge.

Supplemental Information

Supplemental Information includes three figures and Supplemental Experimental Procedures and can be found with this article online at http://dx.doi.org/10.1016/j.cub.2014.08.061.

Acknowledgments

N.S.D., Z.L., R.L., J.F., and S.S. are supported from a Programme Grant to D.M.G. from Cancer Research UK. G.T. is supported from an MRC Programme Grant to D.M.G. M.D. is grateful for a Harmonia 5 Grant (2013/10/M/NZ2/00298) from the Polish National Science Center for collaboration with the D.M.G. lab. Z.L. is grateful for the Federation of European Biochemical Societies for the Long-term post-doctoral Fellowship and R.L. thanks the fellowship support received from Fondazione Italiana per la Ricerca sul Cancro. The authors are thankful for the *Drosophila* Genomics Resource Centre (supported by NIH grant 2P40OD010949-10A1) for the cDNA clones and *Drosophila* gateway destination vectors.

Received: July 18, 2014
Revised: August 13, 2014
Accepted: August 27, 2014
Published: September 25, 2014

References

1. Nigg, E.A., and Raff, J.W. (2009). Centrioles, centrosomes, and cilia in health and disease. Cell *139*, 663–678.
2. O'Connell, K.F., Caron, C., Kopish, K.R., Hurd, D.D., Kemphues, K.J., Li, Y., and White, J.G. (2001). The *C. elegans* zyg-1 gene encodes a regulator of centrosome duplication with distinct maternal and paternal roles in the embryo. Cell *105*, 547–558.
3. Habedanck, R., Stierhof, Y.D., Wilkinson, C.J., and Nigg, E.A. (2005). The Polo kinase Plk4 functions in centriole duplication. Nat. Cell Biol. *7*, 1140–1146.
4. Bettencourt-Dias, M., Rodrigues-Martins, A., Carpenter, L., Riparbelli, M., Lehmann, L., Gatt, M.K., Carmo, N., Balloux, F., Callaini, G., and Glover, D.M. (2005). SAK/PLK4 is required for centriole duplication and flagella development. Curr. Biol. *15*, 2199–2207.
5. Delattre, M., Canard, C., and Gönczy, P. (2006). Sequential protein recruitment in *C. elegans* centriole formation. Curr. Biol. *16*, 1844–1849.
6. Pelletier, L., O'Toole, E., Schwager, A., Hyman, A.A., and Müller-Reichert, T. (2006). Centriole assembly in *Caenorhabditis elegans*. Nature *444*, 619–623.
7. Dzhindzhev, N.S., Yu, Q.D., Weiskopf, K., Tzolovsky, G., Cunha-Ferreira, I., Riparbelli, M., Rodrigues-Martins, A., Bettencourt-Dias, M., Callaini, G., and Glover, D.M. (2010). Asterless is a scaffold for the onset of centriole assembly. Nature *467*, 714–718.
8. Sonnen, K.F., Gabryjonczyk, A.M., Anselm, E., Stierhof, Y.D., and Nigg, E.A. (2013). Human Cep192 and Cep152 cooperate in Plk4 recruitment and centriole duplication. J. Cell Sci. *126*, 3223–3233.
9. Kim, T.S., Park, J.E., Shukla, A., Choi, S., Murugan, R.N., Lee, J.H., Ahn, M., Rhee, K., Bang, J.K., Kim, B.Y., et al. (2013). Hierarchical recruitment of Plk4 and regulation of centriole biogenesis by two centrosomal scaffolds, Cep192 and Cep152. Proc. Natl. Acad. Sci. USA *110*, E4849–E4857.
10. Rodrigues-Martins, A., Riparbelli, M., Callaini, G., Glover, D.M., and Bettencourt-Dias, M. (2007). Revisiting the role of the mother centriole in centriole biogenesis. Science *316*, 1046–1050.
11. Cunha-Ferreira, I., Rodrigues-Martins, A., Bento, I., Riparbelli, M., Zhang, W., Laue, E., Callaini, G., Glover, D.M., and Bettencourt-Dias, M. (2009). The SCF/Slimb ubiquitin ligase limits centrosome amplification through degradation of SAK/PLK4. Curr. Biol. *19*, 43–49.

12. Rogers, G.C., Rusan, N.M., Roberts, D.M., Peifer, M., and Rogers, S.L. (2009). The SCF Slimb ubiquitin ligase regulates Plk4/Sak levels to block centriole reduplication. J. Cell Biol. *184*, 225–239.
13. Kitagawa, D., Busso, C., Flückiger, I., and Gönczy, P. (2009). Phosphorylation of SAS-6 by ZYG-1 is critical for centriole formation in *C. elegans* embryos. Dev. Cell *17*, 900–907.
14. Hatch, E.M., Kulukian, A., Holland, A.J., Cleveland, D.W., and Stearns, T. (2010). Cep152 interacts with Plk4 and is required for centriole duplication. J. Cell Biol. *191*, 721–729.
15. Bahtz, R., Seidler, J., Arnold, M., Haselmann-Weiss, U., Antony, C., Lehmann, W.D., and Hoffmann, I. (2012). GCP6 is a substrate of Plk4 and required for centriole duplication. J. Cell Sci. *125*, 486–496.
16. Sillibourne, J.E., Tack, F., Vloemans, N., Boeckx, A., Thambirajah, S., Bonnet, P., Ramaekers, F.C., Bornens, M., and Grand-Perret, T. (2010). Autophosphorylation of polo-like kinase 4 and its role in centriole duplication. Mol. Biol. Cell *21*, 547–561.
17. Guderian, G., Westendorf, J., Uldschmid, A., and Nigg, E.A. (2010). Plk4 trans-autophosphorylation regulates centriole number by controlling betaTrCP-mediated degradation. J. Cell Sci. *123*, 2163–2169.
18. Holland, A.J., Lan, W., Niessen, S., Hoover, H., and Cleveland, D.W. (2010). Polo-like kinase 4 kinase activity limits centrosome overduplication by autoregulating its own stability. J. Cell Biol. *188*, 191–198.
19. Brownlee, C.W., Klebba, J.E., Buster, D.W., and Rogers, G.C. (2011). The Protein Phosphatase 2A regulatory subunit Twins stabilizes Plk4 to induce centriole amplification. J. Cell Biol. *195*, 231–243.
20. Cunha-Ferreira, I., Bento, I., Pimenta-Marques, A., Jana, S.C., Lince-Faria, M., Duarte, P., Borrego-Pinto, J., Gilberto, S., Amado, T., Brito, D., et al. (2013). Regulation of autophosphorylation controls PLK4 self-destruction and centriole number. Curr. Biol. *23*, 2245–2254.
21. Fu, J., and Glover, D.M. (2012). Structured illumination of the interface between centriole and peri-centriolar material. Open Biol *2*, 120104.
22. Sonnen, K.F., Schermelleh, L., Leonhardt, H., and Nigg, E.A. (2012). 3D-structured illumination microscopy provides novel insight into architecture of human centrosomes. Biol. Open *1*, 965–976.
23. Spektor, A., Tsang, W.Y., Khoo, D., and Dynlacht, B.D. (2007). Cep97 and CP110 suppress a cilia assembly program. Cell *130*, 678–690.
24. Kohlmaier, G., Loncarek, J., Meng, X., McEwen, B.F., Mogensen, M.M., Spektor, A., Dynlacht, B.D., Khodjakov, A., and Gönczy, P. (2009). Overly long centrioles and defective cell division upon excess of the SAS-4-related protein CPAP. Curr. Biol. *19*, 1012–1018.
25. Schmidt, T.I., Kleylein-Sohn, J., Westendorf, J., Le Clech, M., Lavoie, S.B., Stierhof, Y.D., and Nigg, E.A. (2009). Control of centriole length by CPAP and CP110. Curr. Biol. *19*, 1005–1011.
26. Tang, C.J., Fu, R.H., Wu, K.S., Hsu, W.B., and Tang, T.K. (2009). CPAP is a cell-cycle regulated protein that controls centriole length. Nat. Cell Biol. *11*, 825–831.
27. Carvalho-Santos, Z., Machado, P., Alvarez-Martins, I., Gouveia, S.M., Jana, S.C., Duarte, P., Amado, T., Branco, P., Freitas, M.C., Silva, S.T., et al. (2012). BLD10/CEP135 is a microtubule-associated protein that controls the formation of the flagellum central microtubule pair. Dev. Cell *23*, 412–424.
28. Roque, H., Wainman, A., Richens, J., Kozyrska, K., Franz, A., and Raff, J.W. (2012). *Drosophila* Cep135/Bld10 maintains proper centriole structure but is dispensable for cartwheel formation. J. Cell Sci. *125*, 5881–5886.
29. Goshima, G., Wollman, R., Goodwin, S.S., Zhang, N., Scholey, J.M., Vale, R.D., and Stuurman, N. (2007). Genes required for mitotic spindle assembly in *Drosophila* S2 cells. Science *316*, 417–421.
30. Dobbelaere, J., Josué, F., Suijkerbuijk, S., Baum, B., Tapon, N., and Raff, J. (2008). A genome-wide RNAi screen to dissect centriole duplication and centrosome maturation in *Drosophila*. PLoS Biol. *6*, e224.
31. van Breugel, M., Hirono, M., Andreeva, A., Yanagisawa, H.A., Yamaguchi, S., Nakazawa, Y., Morgner, N., Petrovich, M., Ebong, I.O., Robinson, C.V., et al. (2011). Structures of SAS-6 suggest its organization in centrioles. Science *331*, 1196–1199.
32. Kitagawa, D., Vakonakis, I., Olieric, N., Hilbert, M., Keller, D., Olieric, V., Bortfeld, M., Erat, M.C., Flückiger, I., Gönczy, P., and Steinmetz, M.O. (2011). Structural basis of the 9-fold symmetry of centrioles. Cell *144*, 364–375.
33. Strnad, P., Leidel, S., Vinogradova, T., Euteneuer, U., Khodjakov, A., and Gönczy, P. (2007). Regulated HsSAS-6 levels ensure formation of a single procentriole per centriole during the centrosome duplication cycle. Dev. Cell *13*, 203–213.
34. Rodrigues-Martins, A., Bettencourt-Dias, M., Riparbelli, M., Ferreira, C., Ferreira, I., Callaini, G., and Glover, D.M. (2007). DSAS-6 organizes a tube-like centriole precursor, and its absence suggests modularity in centriole assembly. Curr. Biol. *17*, 1465–1472.
35. Stevens, N.R., Dobbelaere, J., Brunk, K., Franz, A., and Raff, J.W. (2010). *Drosophila* Ana2 is a conserved centriole duplication factor. J. Cell Biol. *188*, 313–323.
36. Stevens, N.R., Roque, H., and Raff, J.W. (2010). DSas-6 and Ana2 coassemble into tubules to promote centriole duplication and engagement. Dev. Cell *19*, 913–919.
37. Wang, W.J., Soni, R.K., Uryu, K., and Tsou, M.F. (2011). The conversion of centrioles to centrosomes: essential coupling of duplication with segregation. J. Cell Biol. *193*, 727–739.
38. Tsou, M.F., Wang, W.J., George, K.A., Uryu, K., Stearns, T., and Jallepalli, P.V. (2009). Polo kinase and separase regulate the mitotic licensing of centriole duplication in human cells. Dev. Cell *17*, 344–354.
39. Riparbelli, M.G., Gottardo, M., Glover, D.M., and Callaini, G. (2014). Inhibition of Polo kinase by BI2536 affects centriole separation during *Drosophila* male meiosis. Cell Cycle *13*, 2064–2072.
40. Fong, C.S., Kim, M., Yang, T.T., Liao, J.C., and Tsou, M.F. (2014). SAS-6 assembly templated by the lumen of cartwheel-less centrioles precedes centriole duplication. Dev. Cell *30*, 238–245.

Current Biology, Volume *24*

Supplemental Information

Plk4 Phosphorylates Ana2 to Trigger Sas6 Recruitment and Procentriole Formation

Nikola S. Dzhindzhev, George Tzolovsky, Zoltan Lipinszki, Sandra Schneider, Ramona Lattao, Jingyan Fu, Janusz Debski, Michal Dadlez, and David M. Glover

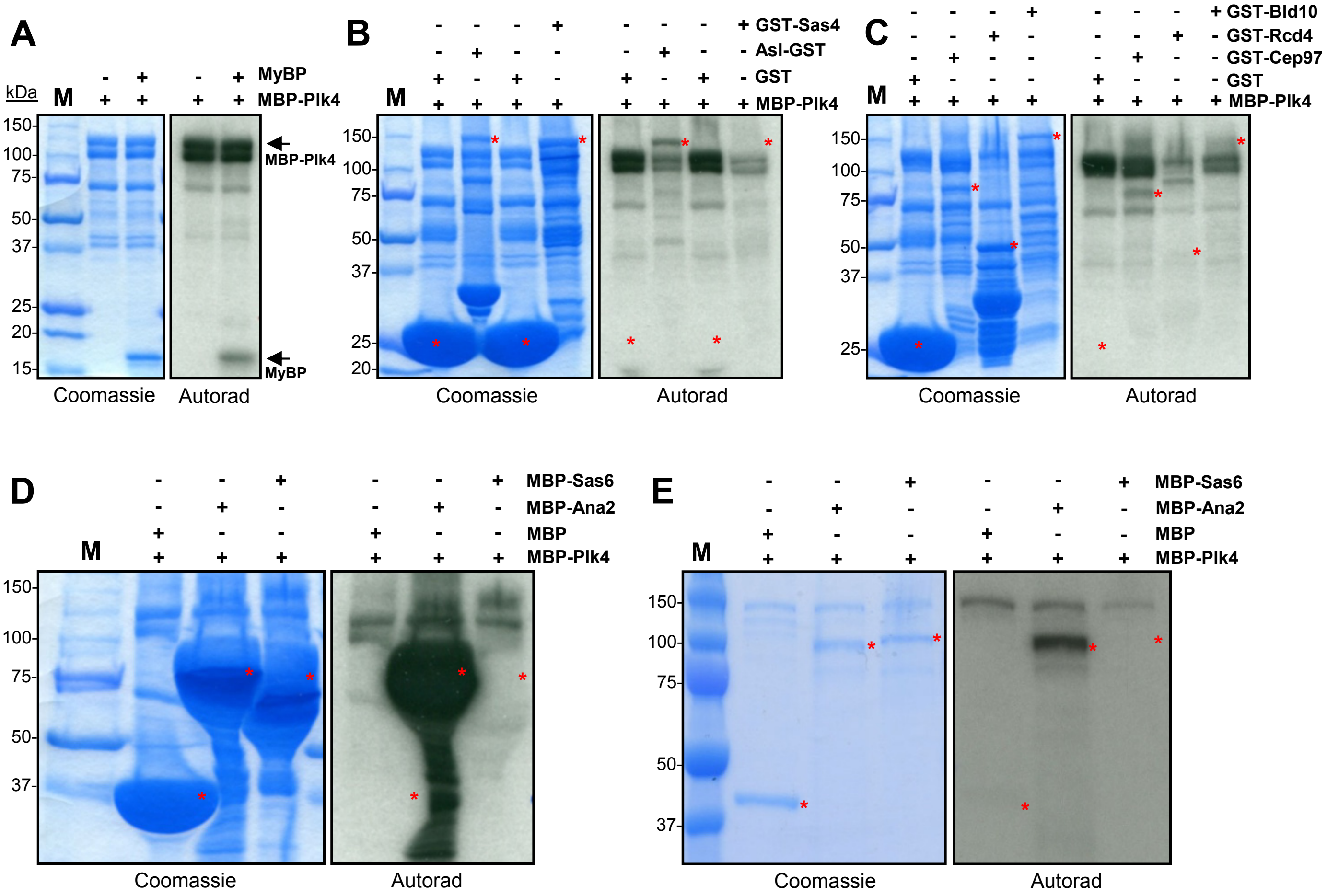

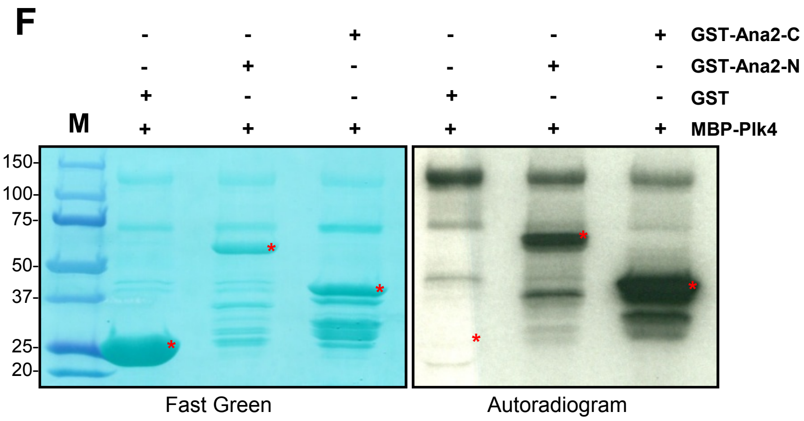

G

Position	*In vitro* Total number of spectral counts	*In vivo* Total number of spectral counts
T69	10	10
S84	13	70
T89	11	0
S98-105	2	15
S119	7	8
T124-125	16	0
S150-153	28	24
T159	62	0
T164	16	0
S177	23	16
S184	2	3
S234	1	3
S237-242	71	9
S318	120	1
S365-373	378	70

Figure S1

Figure S1 (Related to Figure 1): An *in vitro* kinase assay screen identifies Ana2 as a novel substrate of Plk4. (A) Recombinant and constitutively active Maltose Binding Protein-tagged Plk4^{T172E} (MBP-Plk4) was affinity purified and incubated with Myelin Basic Protein (MyBP) as artificial kinase substrate in the presence of ^{32}P-γ-ATP. Following SDS-PAGE and autoradiography it can be seen that MBP-Plk4 phosphorylates MyBP and also autophosphorylates itself demonstrating that the recombinant kinase is active *in vitro*. **(B-E)** Recombinant centriolar proteins tagged with glutathione S-transferase (GST) or MBP, as indicated above the panels, were affinity-purified on the appropriate resins and subsequently incubated with MBP-Plk4 in the presence of ^{32}P-γ-ATP. The reaction mixture was then boiled in Laemmli sample buffer and subjected to SDS-PAGE. Gels were stained with Coomassie Brilliant Blue (left panels), photographed, dried and analysed by autoradiography (right panels). Red asterisks denote the positions of bands corresponding to the full-length recombinant putative substrates. The autoradiograms demonstrate that MBP-Plk4 could phosphorylate Ana2 strongly (**D** and **E**); and Asl (**B**) and Cep97 (**C**) weakly; but could not modify Sas4 (**B**); Bld10 or Rcd4 (**C**); or Sas6 (**D** and **E**). The overloaded gel (**D**) illustrates the strength and specifically of the phosphorylation of Ana2 by MBP-Plk4 *in vitro* as opposed to MBP-alone or MBP-Sas6. Normal loading is shown on panel (**E**). **(F)** *In vitro* kinase assay demonstrating that MBP-Plk4 could phosphorylate both GST-tagged N- terminal part of Ana2 (GST-Ana2-N; residues 1-280 delineated in Figure 1B) as well as the C-terminal part (GST-Ana2-C; residues 281-420, Figure 1B). **(G)** Following phosphorylation by MBP-Plk4 *in vitro*, GST-Ana2 was subjected to phospho-peptide mapping by mass spectrometry. The table (column: *in vitro*) summarises the number of times a particular phospho-peptide was detected. Two tryptic peptides, one containing S318, the other containing S365, S370 and S373 were preferentially phosphorylated by MBP-Plk4 *in vitro*. Protein A-Ana2 was affinity purified from cultured cells also expressing Plk4ND and subjected to phospho-peptide mapping by mass spectrometry. The table (column: *in vivo*) summarises the number of times a particular phospho-peptide was detected. Tryptic peptides containing S318, S365, S370 and S373 within the STAN motif were phosphorylated both *in vitro* and *in vivo*. Several residues identified in our study as phosphorylated by Plk4 were also reported to be phosphorylated in *Drosophila* Kc 167 cells in a phospho-proteome analysis (PhosphoPep [S12]).

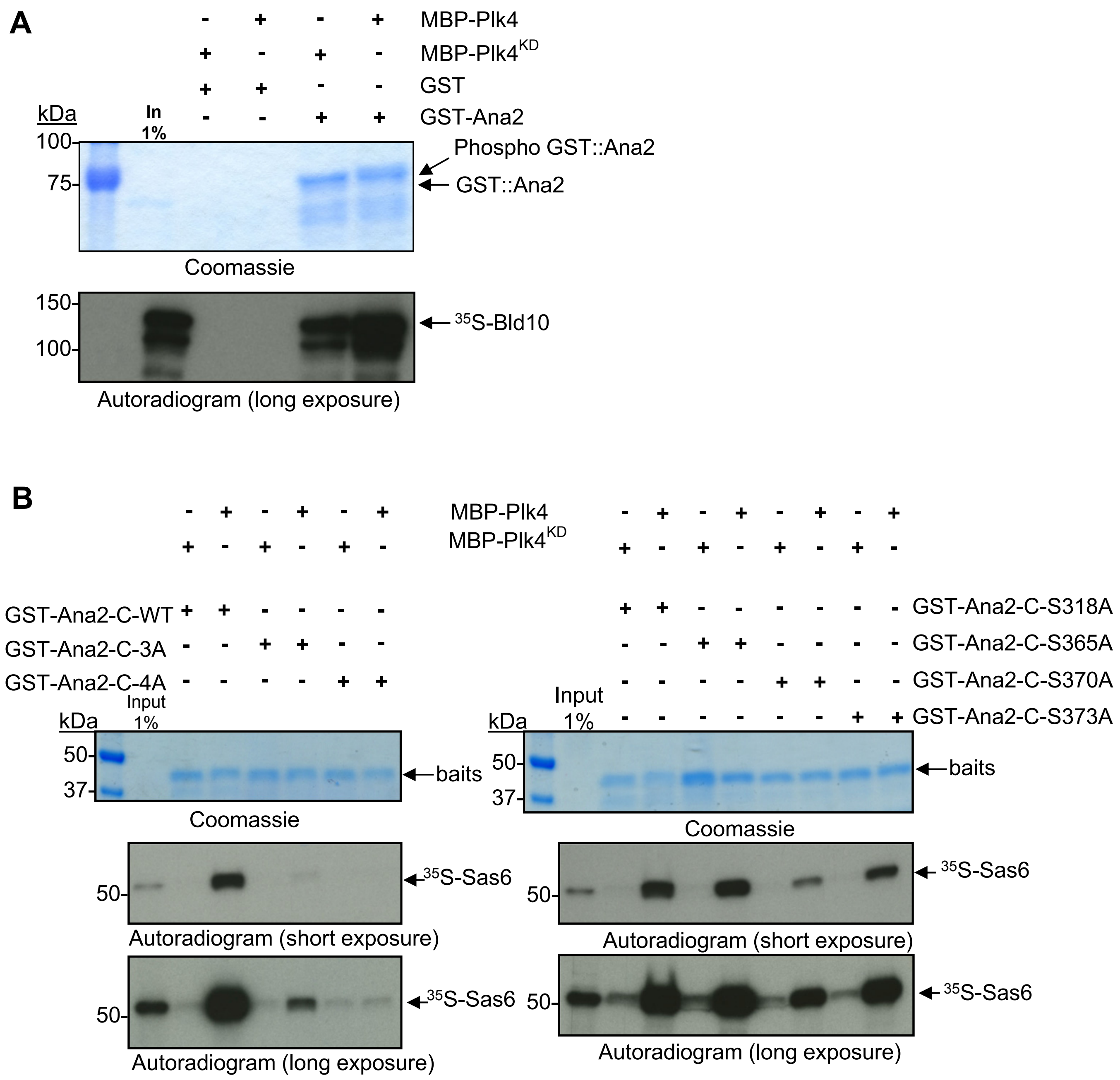
A
MBP-Plk4
MBP-Plk4KD
GST
GST-Ana2
kDa
In 1%
100-
75-
Phospho GST::Ana2
GST::Ana2
Coomassie
150-
100-
35S-Bld10
Autoradiogram (long exposure)
B
MBP-Plk4
MBP-Plk4KD
GST-Ana2-C-WT
GST-Ana2-C-3A
GST-Ana2-C-4A
Input 1%
kDa
50-
37-
baits
Coomassie
50-
35S-Sas6
Autoradiogram (short exposure)
50-
35S-Sas6
Autoradiogram (long exposure)
GST-Ana2-C-S318A
GST-Ana2-C-S365A
GST-Ana2-C-S370A
GST-Ana2-C-S373A
Input 1%
kDa
50-
37-
baits
Coomassie
50-
35S-Sas6
Autoradiogram (short exposure)
50-
35S-Sas6
Autoradiogram (long exposure)

Figure S2

Figure S2 (Related to Figure 2): Plk4-mediated phosphorylation of four conserved serines within the STAN motif of Ana2 is essential for its interaction with Sas6. (A) A longer exposure of the autoradiogram shown in Figure 2A demonstrates that ^{35}S-Met-labelled Bld10 binds both non-phosphorylated and phosphorylated forms of Ana2, *in vitro*. **(B)** Mutation of all 4 conserved Ser-residues within the STAN-motif is required to completely abolish the Plk4-mediated Ana2-Sas6 interaction. ^{35}S-Met-labelled Sas6 was incubated with the following forms of the GST-tagged C-terminal part of Ana2: wild-type (GST-Ana2-C-WT) or with serine to alanine substitutions at residues 365, 370, 373 (GST-Ana2-C-3A), at residues 318, 365, 370, 373 (GST-Ana2-C-4A); or with single S-to-A point mutants at these sites (as indicated). The Ana2 variants were pre-treated with either active or inactive ($Plk4^{KD}$) MBP-tagged Plk4. Complexes were subjected to SDS-PAGE, gels were stained (Coomassie) and analysed by autoradiography. Among the individual serine to alanine substitutions, S370A results in the greatest reduction of Sas6 binding (right-hand panel, penultimate pair of lanes). However, all 4 conserved serine residues need to be mutated to completely disrupt the Ana2-Sas6 interaction (4A-mutant, left-hand panel, final pair of lanes).

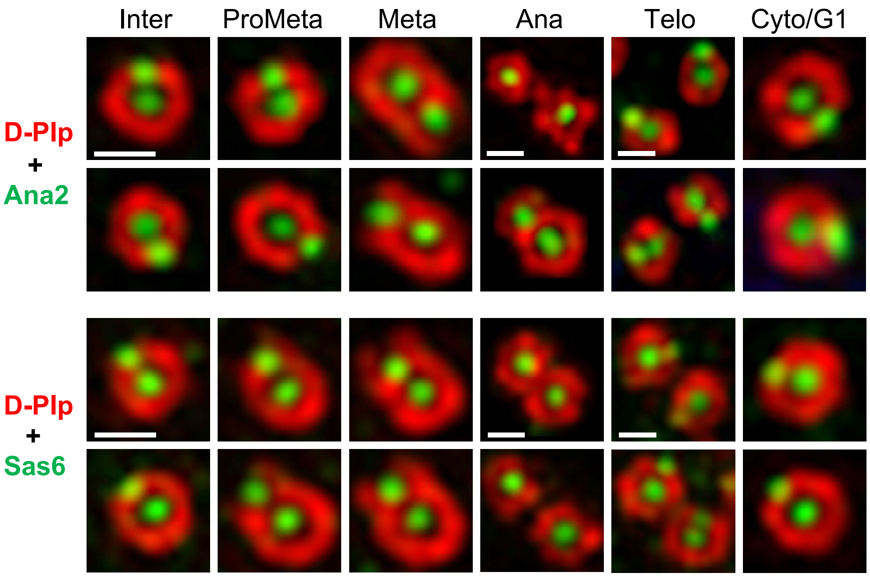

Figure S3

Figure S3 (Related to Figure 3): Centriolar localisation of endogenous Ana2 and Sas6 during the cell cycle. Localisation of endogenous Ana2 and Sas6 relative to D-Plp at the indicated cell cycle stages in D.Mel-2 cells revealed by structured illumination microscopy. At mitotic entry, Ana2 and Sas6 are both present in two discrete puncta, one in the centre of the mother centriole, the other marking the daughter that has yet to be surrounded by D-Plp. After completion of the D-Plp ring of the daughter centriole (metaphase/anaphase) and the disengagement of the mother- daughter pair in anaphase, both centrioles each acquire a new dot of Ana2 or Sas6 at their periphery during late-anaphase/telophase marking the site of the future procentriole formation. Scale bar, 0.5 μm. (Note: in anaphase and telophase the centrioles are shown at lower magnification; Scale bar, 0.5 μm).

Supplemental Experimental Procedures

DNA constructs

cDNA clones for Ana2 (clone LD22033), Sas6 [S1], Plk4 (clone RE70136), Sas4 [S2], Asl (clone GH02902), Cep97 (clone RE26466), Rcd4 (clone SD16838), Cep135/Bld10 (clone LD35990) and Ana1 (LD07765 and IP16240) were obtained from the *Drosophila* Genomics Resource Centre (DGRC), cloned in house or describes previously [S3]. Since no full-length clone for Ana1-RA was available, we created it by using two overlapping partial cDNA clones (LD07765 and IP16240). Full-length Ana2, Sas6, Plk4, Sas4, Asl, Cep97, Rcd4, Cep135/Bld10, Ana1, Ana2-N, Ana2-C and Sas6-C (residues 236-472) were respectively cloned into pDONR221 using the Gateway system (Life Technologies) according to the manufacturer's guidelines. Entry clones were verified by DNA sequencing. Plk4ND and Plk4^{NDKD} entry clones are described elsewhere [S4]. *Drosophila* expression vectors suitable for the *Actin5C* promoter-driven constitutive expression of chimeric proteins with eGFP-, 3xFLAG-, 6xMyc- or Protein A-tags fused to either the amino- or carboxy-terminus of the protein, respectively, were prepared using the following destination vectors obtained from DGRC or made in our lab [S5]: pAFW, pAWF, pAGW, pAWG, pAWM (*Drosophila* Gateway™ Vector Collection (https://emb.carnegiescience.edu/labs/murphy/Gateway%20vectors.html)), pAct5c-PrA (amino-terminal Protein A fusion under the regulation of Actin5c promoter) [S5] and pMT-cPrA (carboxy-terminal Protein A fusion under the regulation of the copper-inducible *Metallothionein A* (*CG9470*) promoter) [S5]. Plasmids for the expression of untagged Ana2-WT, Ana2-4A, Plk4ND and Plk4^{NDKD} were generated by recombining the STOP codon-containing entry clones into pAWF, respectively. Untagged Plk4ND was also made in pMT-cPrA using the same strategy. Due to the stop codon placed between the CDS and carboxy-terminal 3xFLAG- or Protein A-tags, the translated protein does not contain the tags (data not shown). Bacterial expression constructs for amino-terminal GST- or MBP-fusions were made using the following destination vectors: pDEST24 (Life Technologies) and pKM596 (Addgene plasmid 8837); [S6], respectively.

Cell culture, DNA and dsRNA transfections

Culturing of D.Mel-2 cells (Life Technologies) and RNAi techniques were described previously [S3, S5]. DNA transfections were carried out using the FuGENE-HD reagent according to the manufacturer's suggestion (Promega). Selection of stable cell lines was performed as described [S5, S7]. Oligonucleotide primers used to generate dsRNA are listed below or reported elsewhere [S1, S3]:

Ana2-F: 5'-GAATTAATACGACTCACTATAGGGAGAATGTTTGTTCCCGAAACGGAGG-3'
Ana2-R: 5'-GAATTAATACGACTCACTATAGGGAGACAGAGCCGCCAGATCACTCTTA-3'

For the efficient dsRNA-based silencing of endogenous Ana2, the following 225 bp-long hybrid DNA template (which contains the combination of *ana2*'s 5'-UTR and 3'-UTR) was generated by Overlap Extension PCR:

5'-GAATTAATACGACTCACTATAGGGAGACAGATTCTCCCGCTCGAATTTAATTTAATCGGCAAATATAAACAAAT ACGCTCCAAATGTTTGTTCCCGAACCGAAGCTGTTGTGAAAGCATGTACAATGTTCGTTTTGTTATTTATGCATAT GTCTATTTGCGATTTAAGTGGAAATATATTTCAATACACGGAAAAAAATCTCCCTATAGTGAGTCGTATTAATTC-3'

Site-directed mutagenesis

To create point mutants of Plk4 or Ana2, the QuikChange II XL Site-Directed Mutagenesis Kit was applied according to the manufacturer's guidelines (Agilent technologies) using wild-type cDNA or entry clones as template and the following oligonucleotide primers:

Plk4-T172E-F: 5'-AGCGACCTGATGAGCGCCATATGGAGATGTGTGGAACTCCGAACTATAT-3'
Plk4-T172E-R: 5'-ATATAGTTCGGAGTTCCACACATCTCCATATGGCGCTCATCAGGTCGCT-3'
Plk4-K43M-F: 5'-ACACTCACCAGGATGTGGCCATAATGATGATCGATAAAAAACTAATCCA-3'
Plk4-K43M-R: 5'-TGGATTAGTTTTTTATCGATCATCATTATGGCCACATCCTGGTGAGTGT-3'
Ana2-S318A-F: 5'-ACTGGCCAAGCCCAACACCGAGAAGGCAATGGTGATGAACGAGCTGGCGCTGA-3'
Ana2-S318A-R: 5'-TCAGCGCCAGCTCGTTCATCACCATTGCCTTCTCGGTGTTGGGCTTGGCCAGT-3'
Ana2-S365A-F: 5'-GATCGACAACATAGGCCACGCGCAGGCACCAAACGACATATCCAATGCTTCGT-3'
Ana2-S365A-R: 5'-ACGAAGCATTGGATATGTCGTTTGGTGCCTGCGCGTGGCCTATGTTGTCGATC-3'
Ana2-S370A-F: 5'-CCACGCGCAGAGTCCAAACGACATAGCAAATGCTTCGTACAAGTATCTCAAAA-3'
Ana2-S370A-R: 5'-TTTTGAGATACTTGTACGAAGCATTTGCTATGTCGTTTGGACTCTGCGCGTGG-3'
Ana2-S373A-F: 5'-GAGTCCAAACGACATATCCAATGCTGCATACAAGTATCTCAAAAAATACCGTC-3'
Ana2-S373A-R: 5'-GACGGTATTTTTTGAGATACTTGTATGCAGCATTGGATATGTCGTTTGGACTC-3'
Ana2-3A-F: 5'-CCACGCGCAGGCACCAAACGACATAGCAAATGCTGCATACAAGTATCTCAAAAAATACCGTC-3'
Ana2-3A-R: 5'-GACGGTATTTTTTGAGATACTTGTATGCAGCATTTGCTATGTCGTTTGGTGCCTGCGCGTGG-3'

Ana2-4A mutant was generated by using *Ana2-S318A-F and R* oligonucleotide primers and Ana2-3A entry clone as template.

Recombinant protein expression and purification

To obtain recombinant constitutively active Plk4 (MBP-Plk4), the activating T172E mutation was introduced into Plk4's CDS, while for the kinase-dead version (MBP-$Plk4^{KD}$), both T172E and the inactivating K43M mutations were introduced into the Plk4 sequence according to [S8]. The recombinant proteins were expressed in *E. coli* strain BL21 Star (DE3)pLysS (Life Technologies) following standard procedures and subsequently purified on Amylose resin (New England Biolabs) according to the manufacturer's instructions. The fusion proteins were eluted with Plk4 kinase buffer (Plk4-KB: 20 mM Na-HEPES pH 7.5, 100 mM NaCl, 10 mM $MgCl_2$, 10 mM $MnCl_2$, 1 mM DTT), supplemented with 15 mM D-Maltose. The quality of purified enzymes was analysed by SDS-PAGE. The concentration was estimated by spectrophotometry. Purified enzymes were stored at -80°C in Plk4-KB supplemented with 50% glycerol. MBP- and GST-tagged recombinant centrosomal proteins used in this study were expressed and purified as described previously [S3].

Protein purification from *Drosophila* cultured cells for phospho-residue mapping

For the affinity purification-coupled mass spectrometric analysis of Ana2 *in vivo* phosphorylation, stably transfected D.Mel-2 cells co-expressing Protein A-Ana2 (constitutive expression) and $Plk4^{ND}$ (expression induced by 0.5 mM $CuSO_4$ for 16 h) transgenic proteins were treated with 100 nM okadaic acid (OA, Calbiochem) for 2 h. Then, cells were harvested and whole cell lysate was prepared in extraction buffer [S9] supplemented with 100 nM OA and phosphatase inhibitor cocktail 2 and 3 (Sigma) as described previously [S5]. Detailed protocol for Protein A affinity purification and sample processing can be found in [S5, S9].

***In vitro* Plk4 kinase assay**

Typically, 4-10 μg of recombinant MBP-Plk4 or MBP-$Plk4^{KD}$ were used in a 20-50 μl reaction volume for 30-60 minutes at 30 °C. For non-radioactive kinase assays substrates on beads were incubated with the enzyme in Plk4-KB supplemented with 250 μM ATP, while for radioactive kinase assay, the phosphorylation took place in the presence of 10 μM ATP and approximately 0.2 MBq of ^{32}P-γ-ATP.

Mass-spectrometry and phospho-peptide/residue mapping

Plk4-pre-phosphorylated recombinant GST-Ana2 or transgenic Protein A-Ana2 proteins were affinity-purified and digested with trypsin. Phosphorylated peptides were isolated using titanium dioxide, followed by LC/MS analysis as detailed in [S10].

***In vitro* binding assay**

Affinity-purified GST, GST-Ana2 (WT or 4A), GST-Ana2-N, GST-Ana2-C or point mutants of GST-Ana2-C were kept on Glutathione Sepharose 4B resin (GE Healthcare) and used as substrates for Plk4 and bait in binding assays. Bait proteins (~4-5 µg) were treated with MBP-Plk4 or MBP-Plk4^{KD} as explained above. Then the baits were supplemented with 600 µl binding buffer (50 mM Na-HEPES pH 7.5, 100 mM NaCl, 2 mM $MgCl_2$, 1 mM EGTA, 1 mM DTT, 0.1 % Triton-X 100, PhosStop (Roche), EDTA-free complete protease inhibitor cocktail (Roche), 100 nM okadaic acid (Calbiochem) and 0.5 mg/ml BSA) and incubated for 2 h at 4 °C with 15 µL ^{35}S-Met-labelled Sas6. ^{35}S-Sas6 was expressed in coupled *in vitro* transcription/translation reaction according to [S3]. After several washes with binding buffer (without BSA) beads were settled, boiled in 2x Laemmli sample buffer and subjected to SDS-PAGE followed by Coomassie Brilliant Blue staining. Gels were scanned, dried and used for autoradiography.

Co-immunoprecipitation

D.Mel-2 cells were transiently co-transfected with Actin5C-promoter-driven constructs encoding 3xFLAG-Ana2 (WT or 4A), Sas6-6xMyc or Plk4 (ND or NDKD) for 22 h. Cell were treated with 25 µM MG132 (Sigma) for 2.5 h, harvested and lysed in 1 ml ice-cold buffer containing 20 mM Tris–HCl pH 7.5, 150 mM NaCl, 2 mM $MgCl_2$,0.5 mM Na-EGTA pH 8.0, 1 mM DTT, 0.1 % NP-40, 5 % glycerol, 1 mM PMSF, EDTA-free complete protease inhibitor cocktail, Roche, supplemented with 100 nM okadaic acid and PhosStop (Roche) by 6x passing through a pre-chilled G25 needle. Lysates were clarified by centrifugation (4 °C, 10,000 g, 10 min) and supernatants were mixed with anti-FLAGM_2 magnetic beads (Sigma) for 3 h at 4 °C. After several washes proteins were eluted with 150 µg/ml 3xFLAG peptide, mixed with Laemmli sample buffer and subjected to SDS-PAGE followed by immunoblotting using standard procedures.

Antibodies

MBP-Ana2 (full length) and GST-Sas6-C (residues 236-472) were expressed and purified from *E. coli* and used as antigens for immunization of rats (Animal Facility at Instituto de Biologia Molecular e Celular, Porto, Portugal). GST-Ana2-N (residues 1-280) was used for production of a rabbit polyclonal antibody (Harlan Laboratories). GST-Plk4$^{301\text{-}600}$ and GST-Plk4$^{601\text{-}769}$ were expressed and purified from *E.coli*, combined and used to immunize sheep (Scottish National Blood Transfusion Service). Rabbit and sheep immunoglobulins were purified on Protein A or G sepharose according to the manufacturer's suggestion (GE Healthcare). We used the following primary antibodies for immuno-fluorescence (IF) or western blotting (WB): Rat-anti-Ana2 (this study; WB 1:4,000); Rabbit-anti-Asl [S3], WB 1:2,000; Sheep-anti-Plk4 (this study; WB 1:2,000); Rabbit-anti-Ana2 (this study; IF 1:1,000); Rat-anti-Sas6 (this study; IF 1:1,000); Chicken-anti-D-Plp [S2] IF 1:1,000; Mouse-anti-α-tubulin (clone DM1A Sigma, WB 1:5,000); Mouse-anti-FLAG (clone M2, Sigma, WB 1:20,000); Mouse-anti-Myc (clone 9E10, Abcam, WB 1:5,000).

Immunostaining and fluorescence microscopy

These procedures were described here [S3]. Briefly, D.Mel-2 cells were grown on concanavalinA-coated coverslips for 2–4h and fixed in cold methanol. Blocking (10% foetal calf serum), antibody incubations and washes were done in PBS, supplemented with 0.1% Triton X-100. Cells were mounted in Vectashield media with DAPI (Vector laboratories). Microscopic analysis was performed on a Carl Zeiss Axiovert 200M microscope with 40x/1; 63x/1.25 and 100x/1.4 Plan Apochromat objectives. Images were acquired with a Photometrics Cool SNAP HQ2 camera and the image analysis software Metamorph (v7.7).

Structured illumination microscopy:

Super-resolution images were acquired on an OMX-V3 system using a 63x/1.4NA oil Olympus lens as described previously [S11]. Images (512x512 ppi) were reconstructed and registered using the SoftWorx Linux package. Images were further processed to obtain maximum intensity projections. These were cropped and assembled in Photoshop v6.

Supplemental references

S1. Rodrigues-Martins, A., Bettencourt-Dias, M., Riparbelli, M., Ferreira, C., Ferreira, I., Callaini, G., and Glover, D.M. (2007). DSAS-6 organizes a tube-like centriole precursor, and its absence suggests modularity in centriole assembly. Current biology : CB *17*, 1465-1472.

S2. Rodrigues-Martins, A., Riparbelli, M., Callaini, G., Glover, D.M., and Bettencourt-Dias, M. (2007). Revisiting the role of the mother centriole in centriole biogenesis. Science *316*, 1046-1050.

S3. Dzhindzhev, N.S., Yu, Q.D., Weiskopf, K., Tzolovsky, G., Cunha-Ferreira, I., Riparbelli, M., Rodrigues- Martins, A., Bettencourt-Dias, M., Callaini, G., and Glover, D.M. (2010). Asterless is a scaffold for the onset of centriole assembly. Nature *467*, 714-718.

S4. Cunha-Ferreira, I., Rodrigues-Martins, A., Bento, I., Riparbelli, M., Zhang, W., Laue, E., Callaini, G., Glover, D.M., and Bettencourt-Dias, M. (2009). The SCF/Slimb ubiquitin ligase limits centrosome amplification through degradation of SAK/PLK4. Current biology : CB *19*, 43-49.

S5. D'Avino, P.P., Archambault, V., Przewloka, M.R., Zhang, W., Laue, E.D., and Glover, D.M. (2009). Isolation of protein complexes involved in mitosis and cytokinesis from Drosophila cultured cells. Methods in molecular biology (Clifton, N.J.) 545, 99-112.

S6. Fox, J.D., Routzahn, K.M., Bucher, M.H., and Waugh, D.S. (2003). Maltodextrin-binding proteins from diverse bacteria and archaea are potent solubility enhancers. FEBS Lett *537*, 53-57.

S7. D'Avino, P.P., Savoian, M.S., Capalbo, L., and Glover, D.M. (2006). RacGAP50C is sufficient to signal cleavage furrow formation during cytokinesis. J Cell Sci *119*, 4402-4408.

S8. Swallow, C.J., Ko, M.A., Siddiqui, N.U., Hudson, J.W., and Dennis, J.W. (2005). Sak/Plk4 and mitotic fidelity. Oncogene *24*, 306-312.

S9 Lipinszki, Z., Wang, P., Grant, R., Lindon, C., Dzhindzhev, N.S., D'Avino, P.P., Przewloka, M.R., Glover, D.M., and Archambault, V. (2014). Affinity purification of protein complexes from Drosophila embryos in cell cycle studies. Methods in molecular biology (Clifton, N.J.) 1170, 571-588.

S10 Graczyk, D., Debski, J., Muszynska, G., Bretner, M., Lefebvre, O., and Boguta, M. (2011). Casein kinase II-mediated phosphorylation of general repressor Maf1 triggers RNA polymerase III activation. Proceedings of the National Academy of Sciences of the United States of America *108*, 4926-4931.

S11. Fu, J., and Glover, D.M. (2012). Structured illumination of the interface between centriole and peri- centriolar material. Open biology *2*, 120104.

S12. Bodenmiller, B., Malmstrom, J., Gerrits, B., Campbell, D., Lam, H., Schmidt, A., Rinner, O., Mueller, L.N., Shannon, P.T., Pedrioli, P.G., et al. (2007). PhosphoPep--a phosphoproteome resource for systems biology research in Drosophila Kc167 cells. Molecular systems biology *3*, 139.
